# Supplementary figures and images for: Reprogramming of GM-CSF-dependent alveolar macrophages through GSK3 activity modulation
Source: eLife. 2025 May 14;14:RP102659. doi: 10.7554/eLife.102659 (PMC12077879; doi:10.7554/eLife.102659)

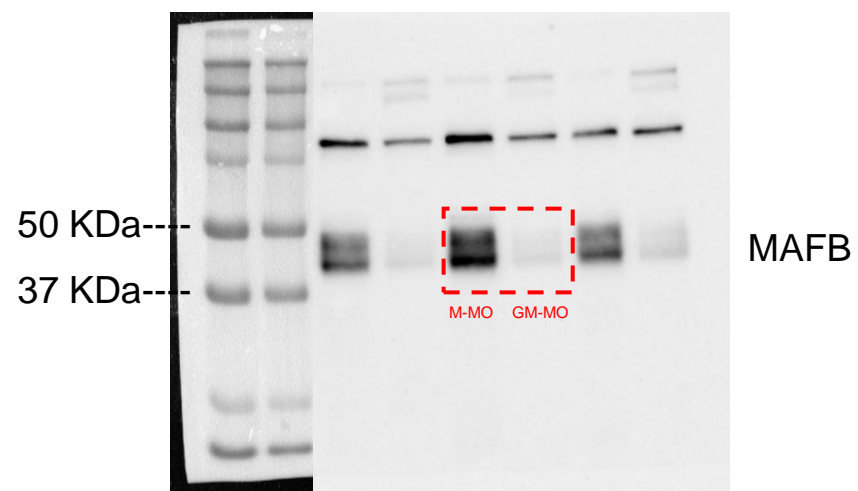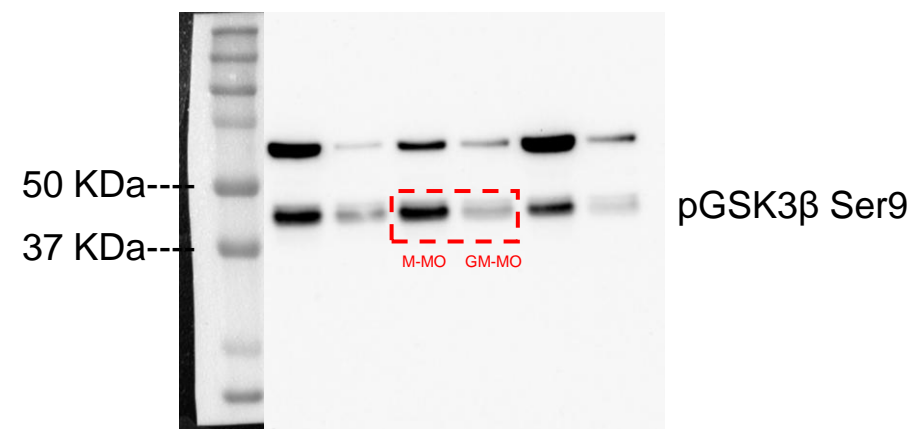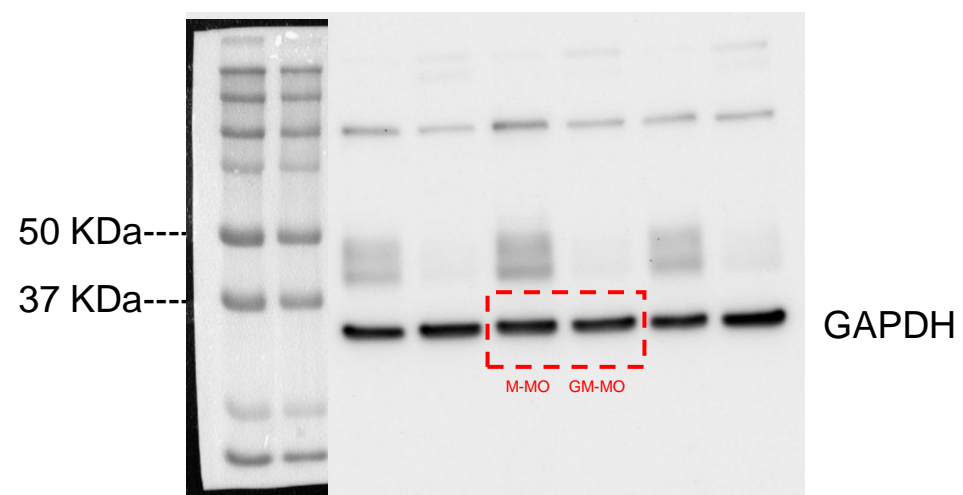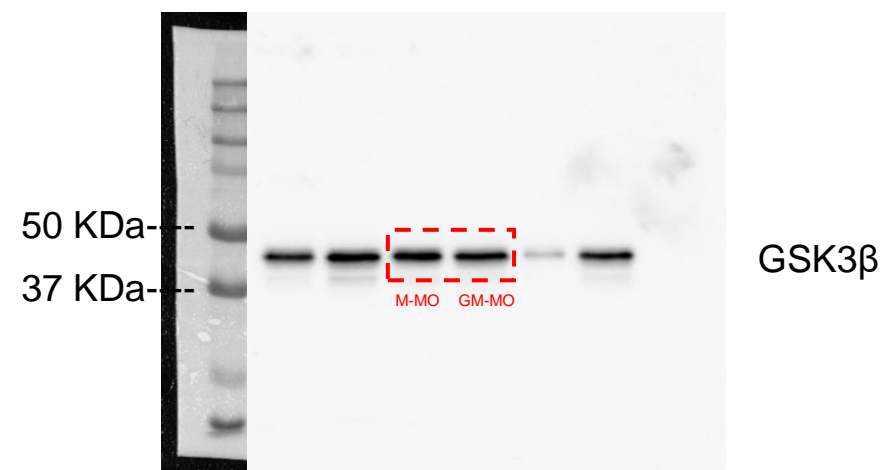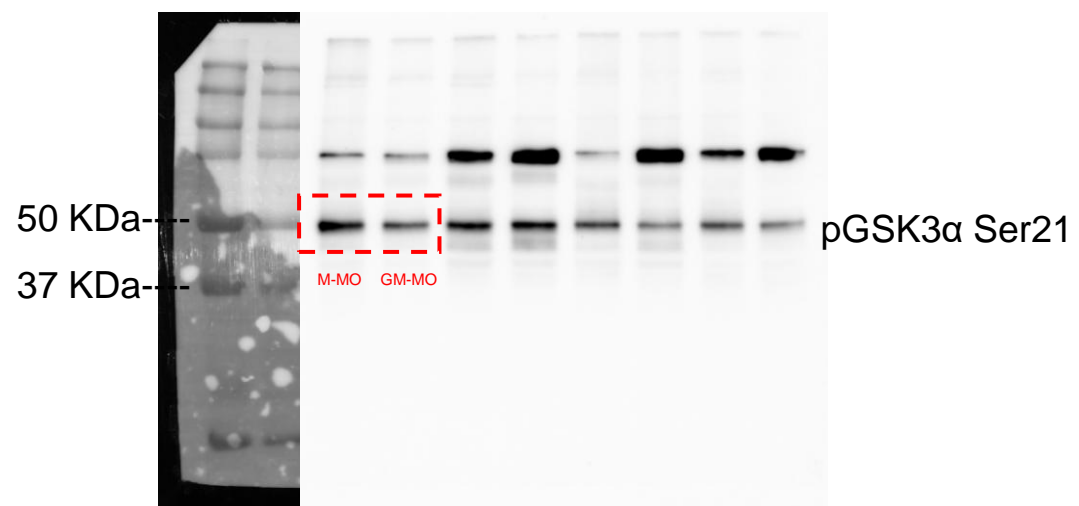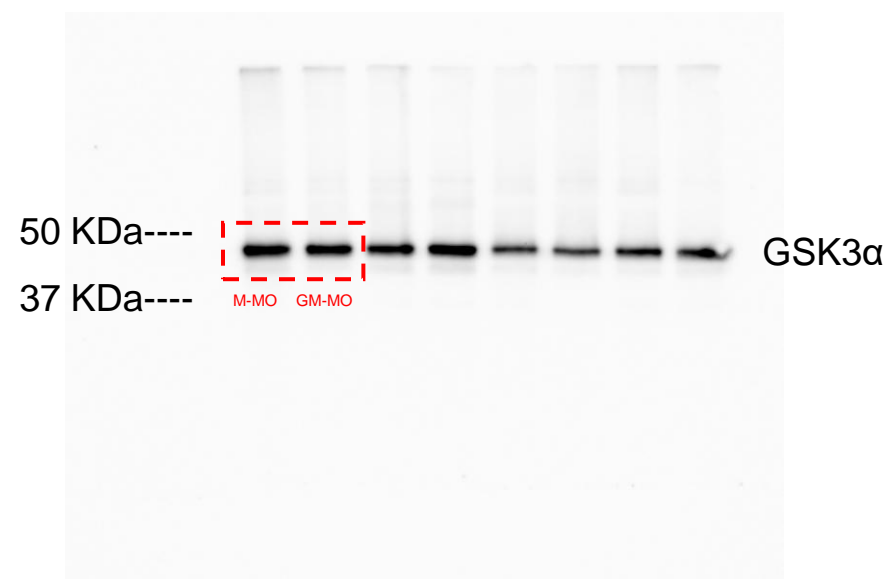

Figure 1A

Supplement: Figure 1—source data 1. [file elife-102659-fig1-data1.pdf]

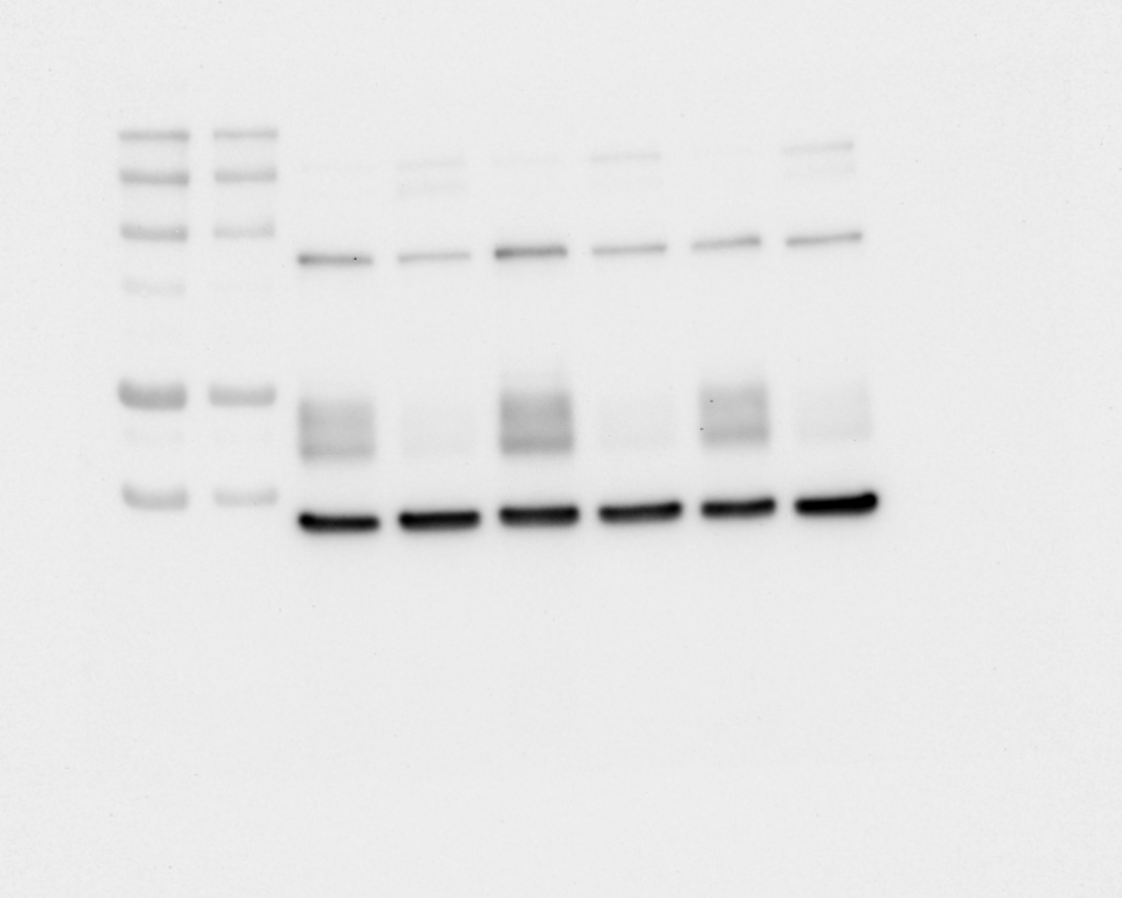

Supplement: Figure 1—source data 2. [file elife-102659-fig1-data2.zip › Figure 1 - Source data 2/GAPDH.tif]

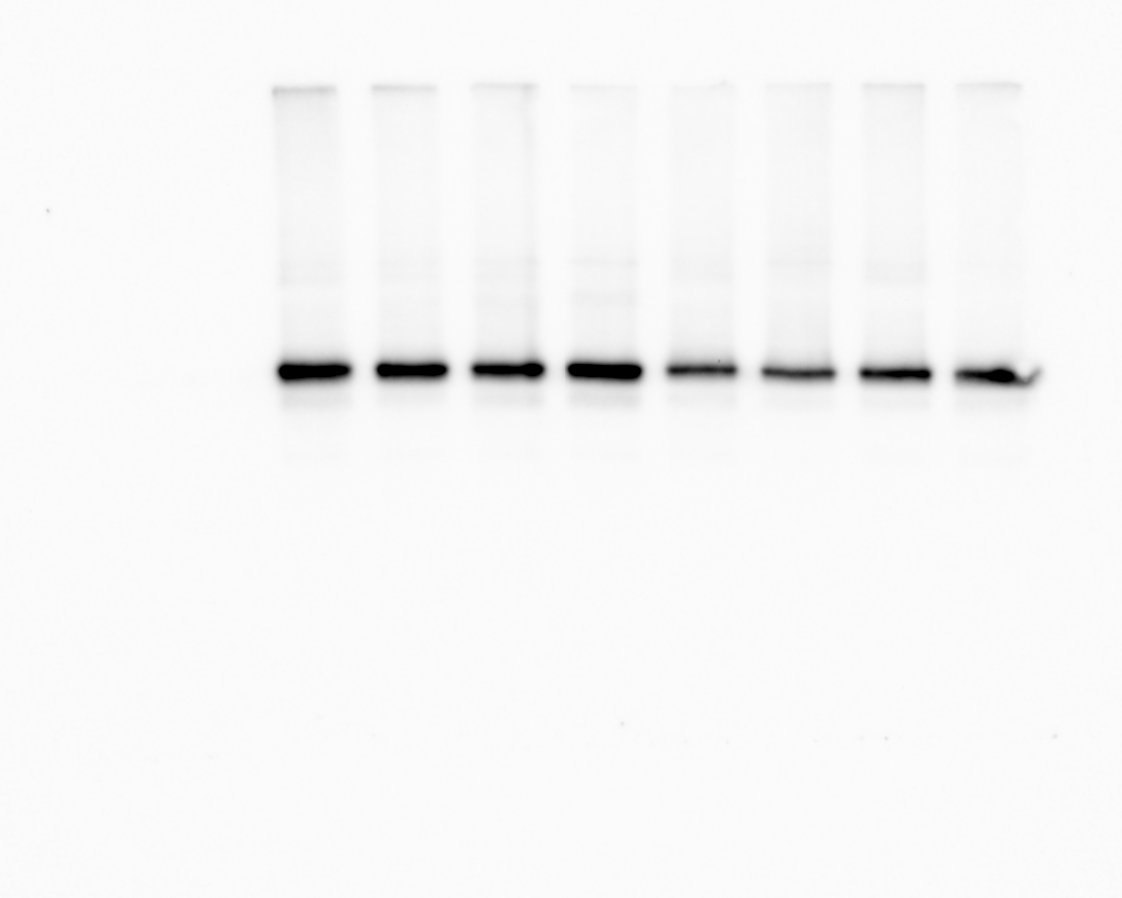

Supplement: Figure 1—source data 2. [file elife-102659-fig1-data2.zip › Figure 1 - Source data 2/GSK3a.tif]

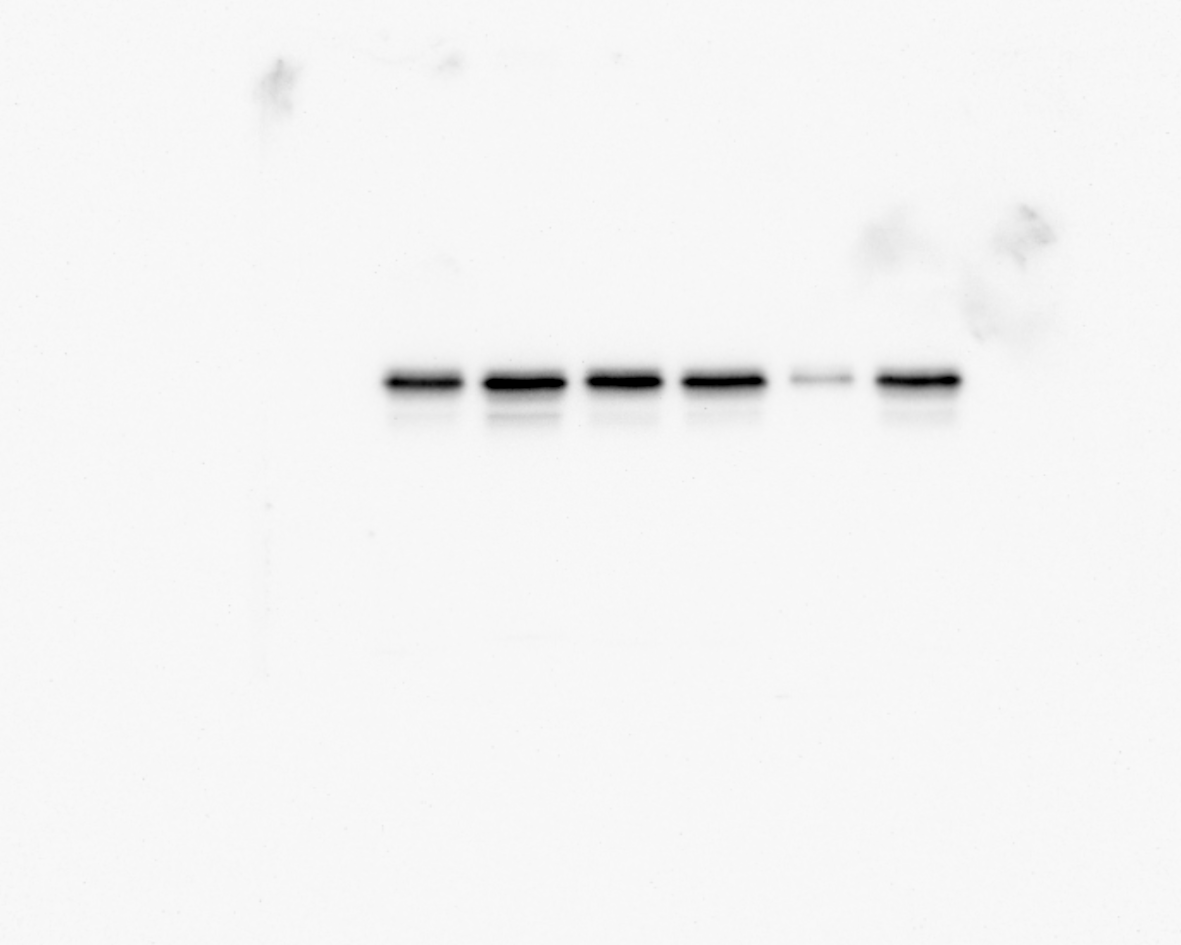

Supplement: Figure 1—source data 2. [file elife-102659-fig1-data2.zip › Figure 1 - Source data 2/GSK3b.tif]

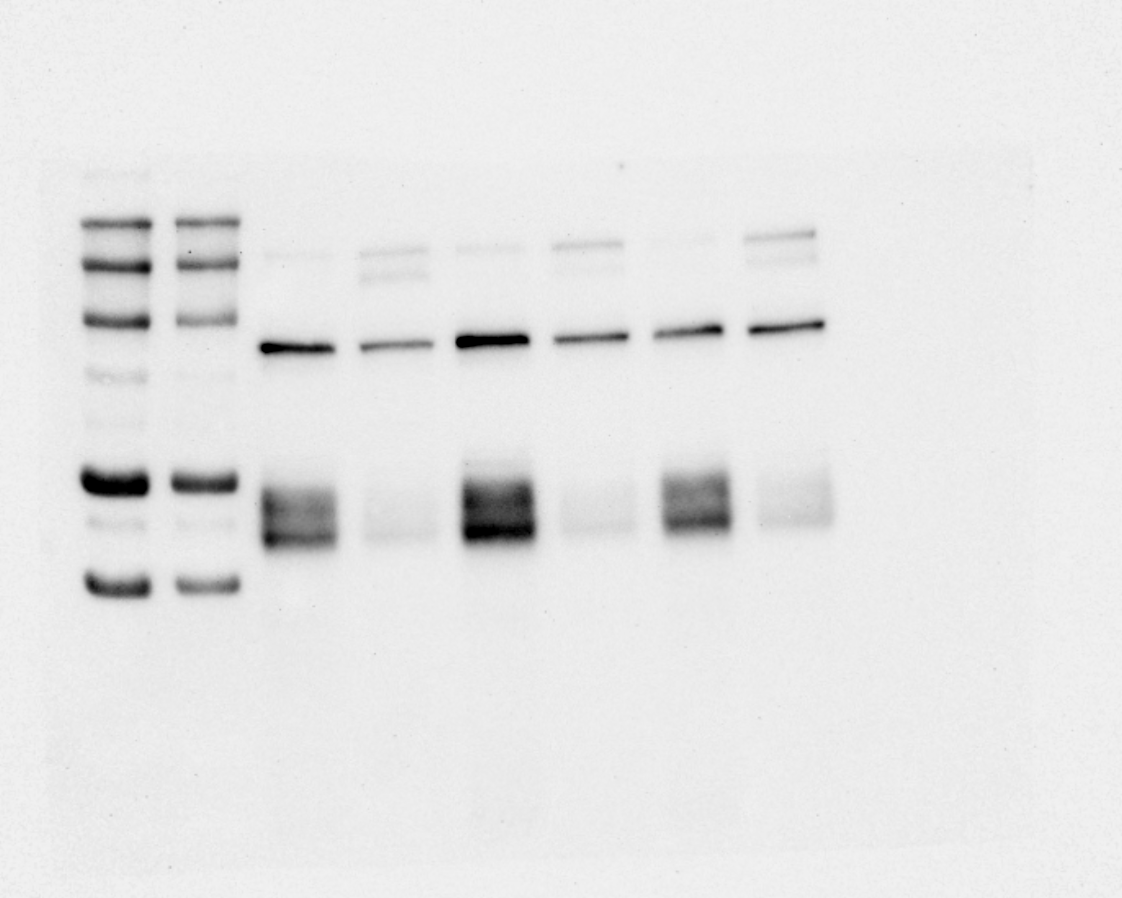

Supplement: Figure 1—source data 2. [file elife-102659-fig1-data2.zip › Figure 1 - Source data 2/MAFB.tif]

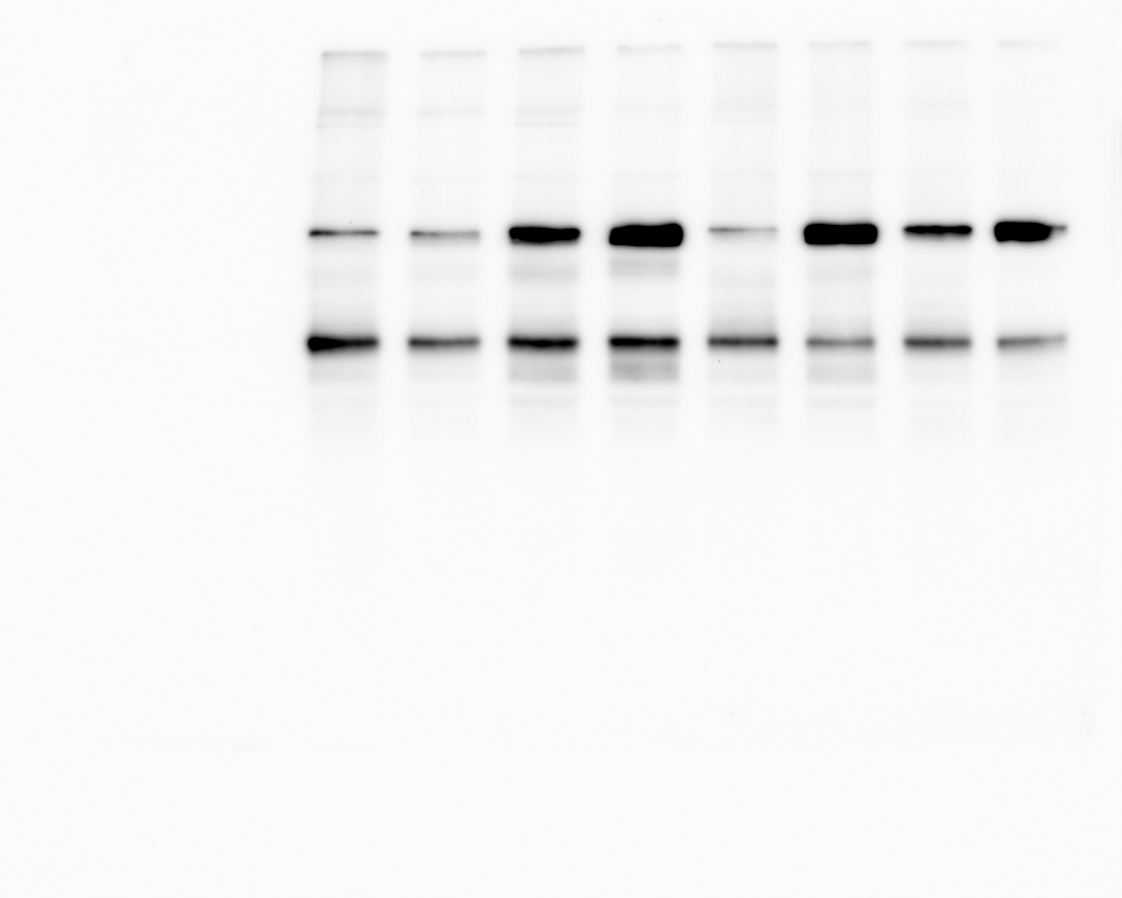

Supplement: Figure 1—source data 2. [file elife-102659-fig1-data2.zip › Figure 1 - Source data 2/p-Ser21-GSK3b.tif]

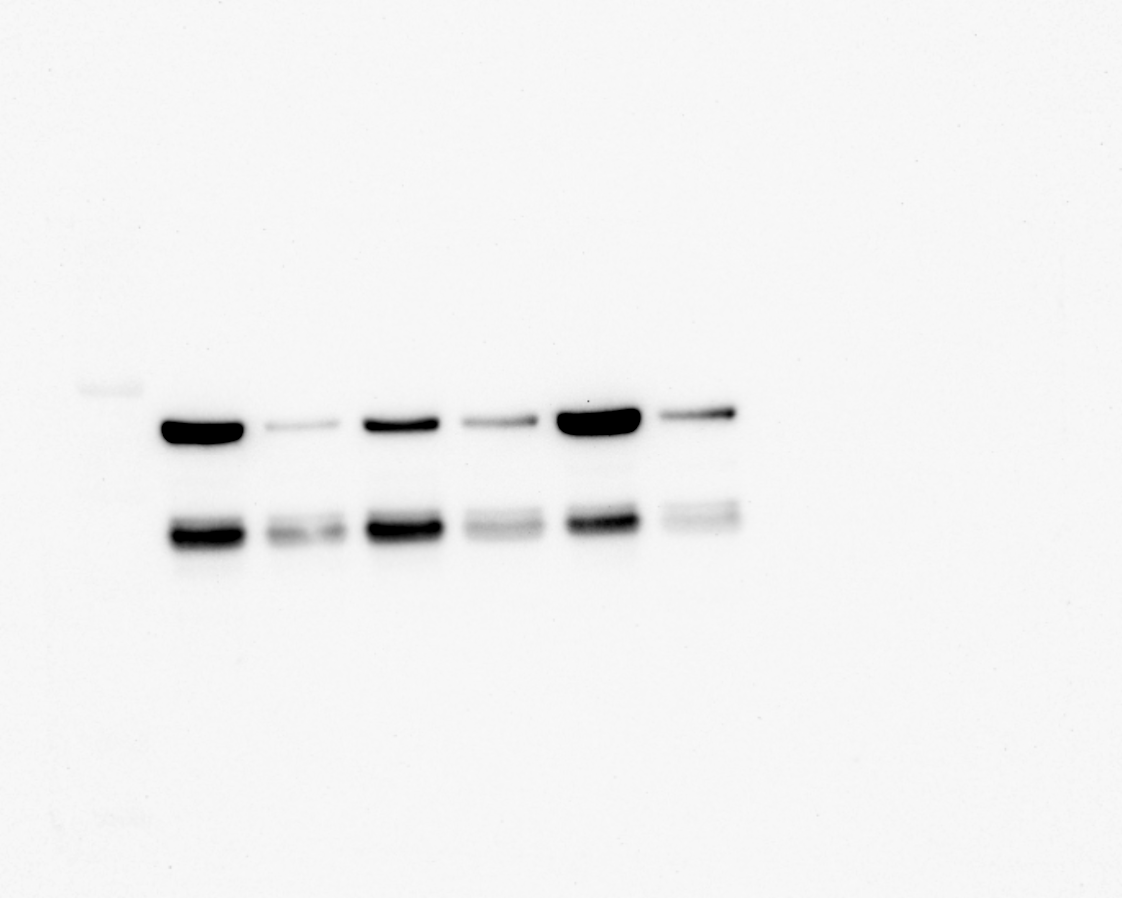

Supplement: Figure 1—source data 2. [file elife-102659-fig1-data2.zip › Figure 1 - Source data 2/p-Ser9-GSK3b.tif]

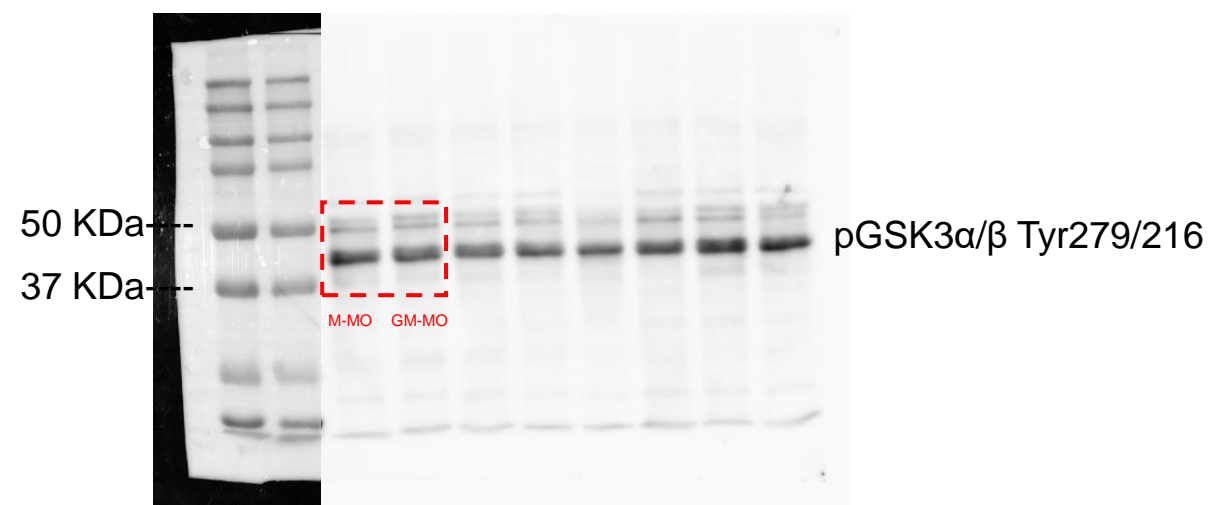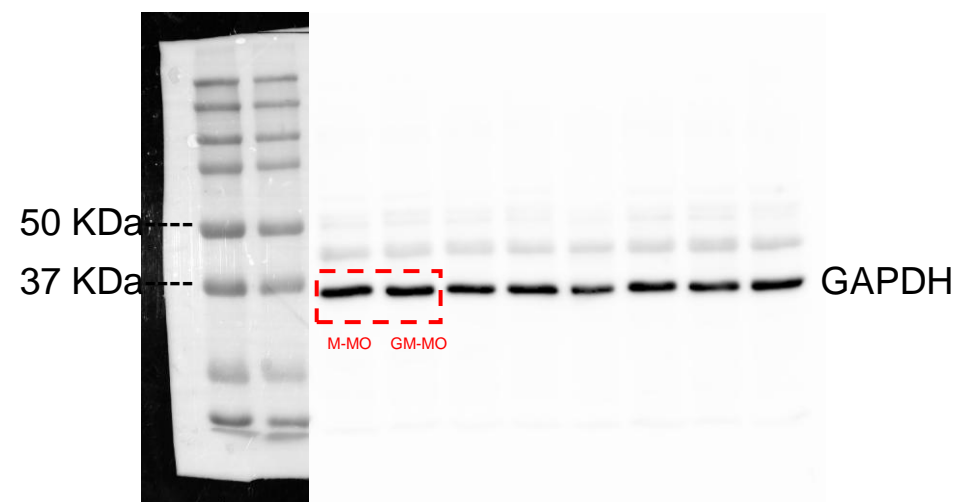

Figure 1B

Supplement: Figure 1—source data 4. [file elife-102659-fig1-data4.pdf]

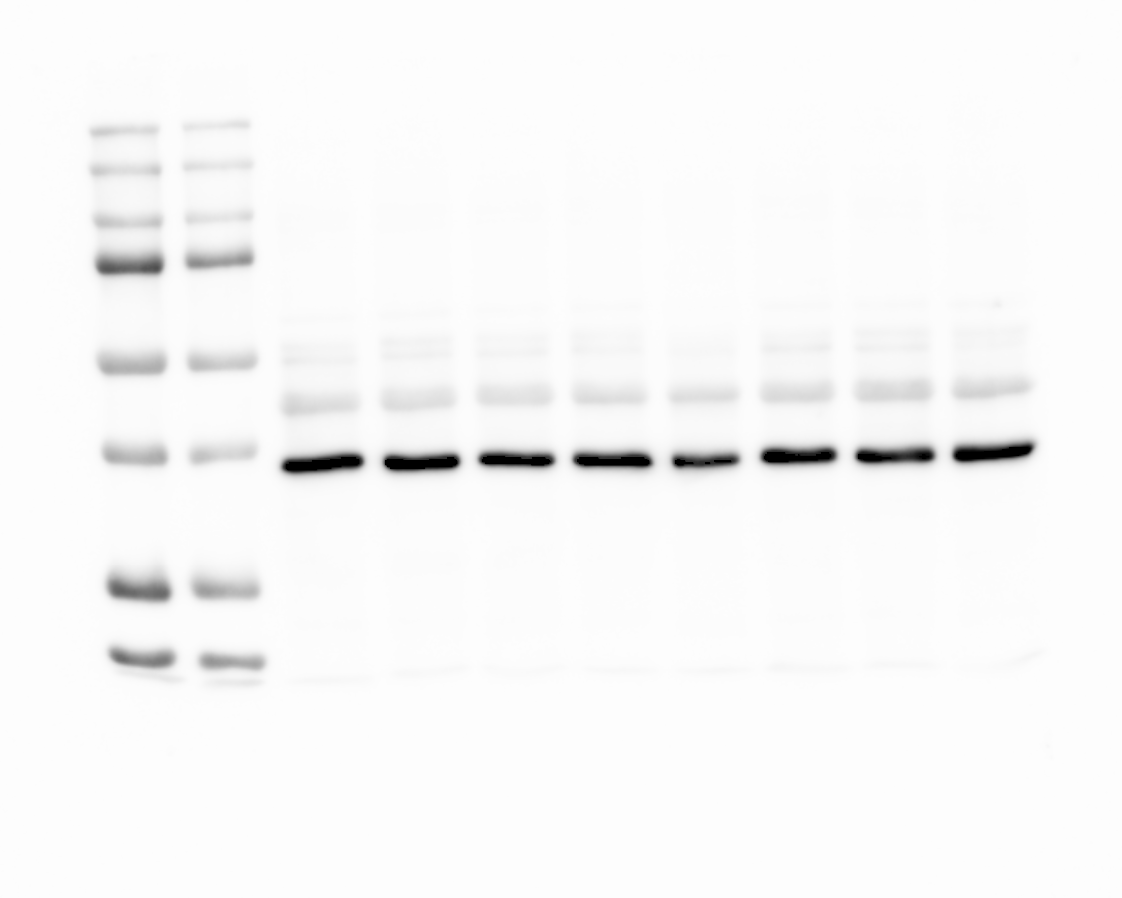

Supplement: Figure 1—source data 5. [file elife-102659-fig1-data5.zip › Figure 1 - Source data 5/GAPDH.tif]

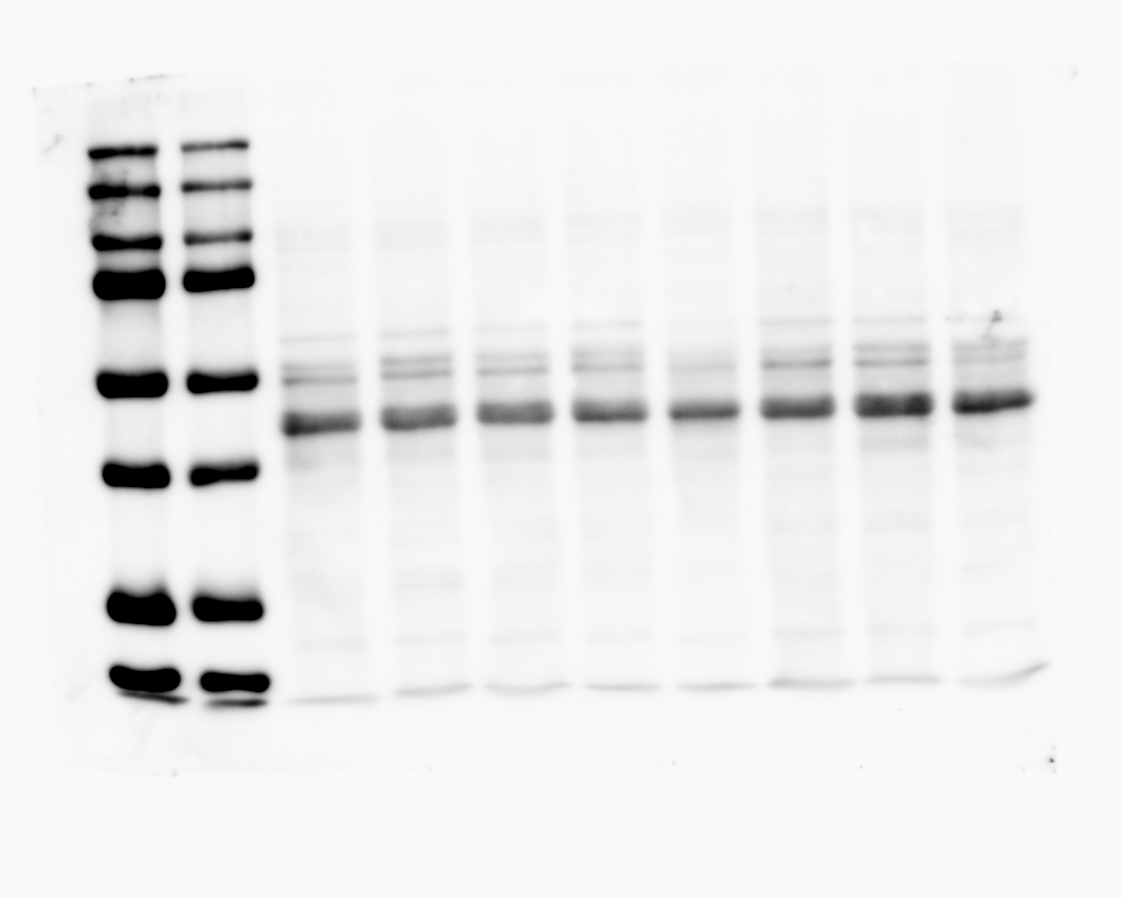

Supplement: Figure 1—source data 5. [file elife-102659-fig1-data5.zip › Figure 1 - Source data 5/p-Tyr279-GSK3a and p-Tyr216-GSK3b.tif]

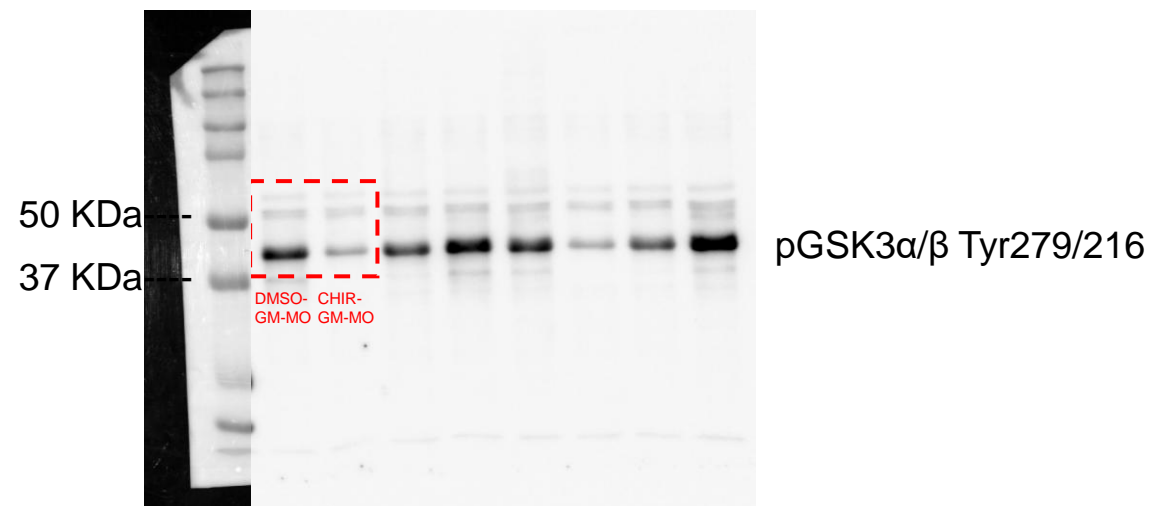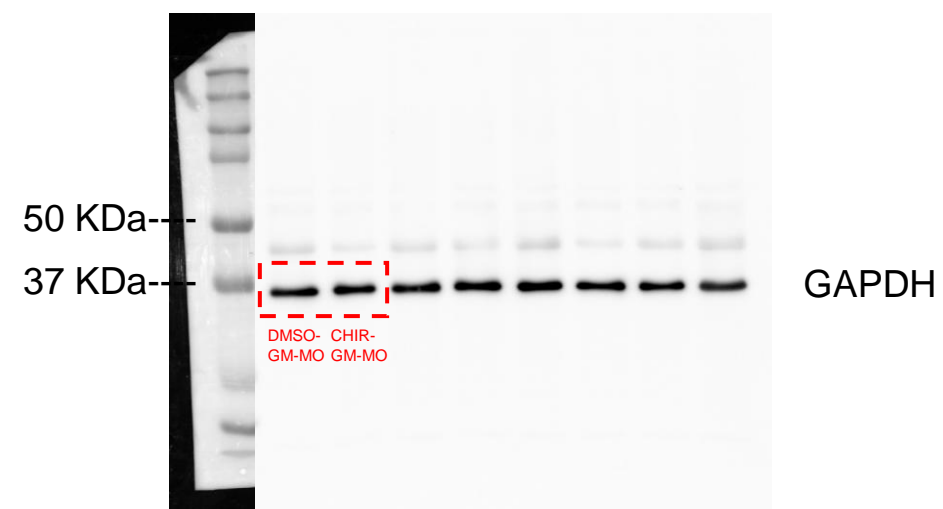

Figure 1D

Supplement: Figure 1—source data 7. [file elife-102659-fig1-data7.pdf]

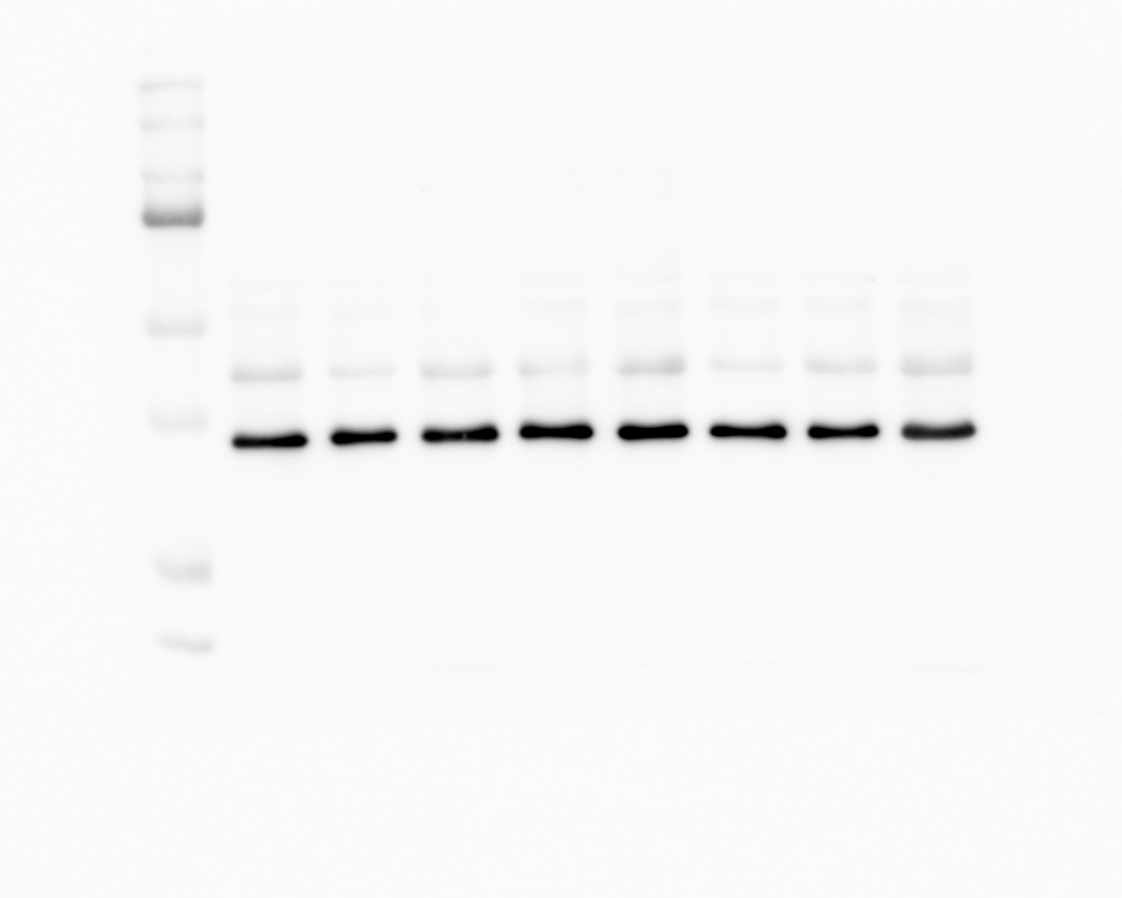

Supplement: Figure 1—source data 8. [file elife-102659-fig1-data8.zip › Figure 1 - Source data 8/GAPDH.tif]

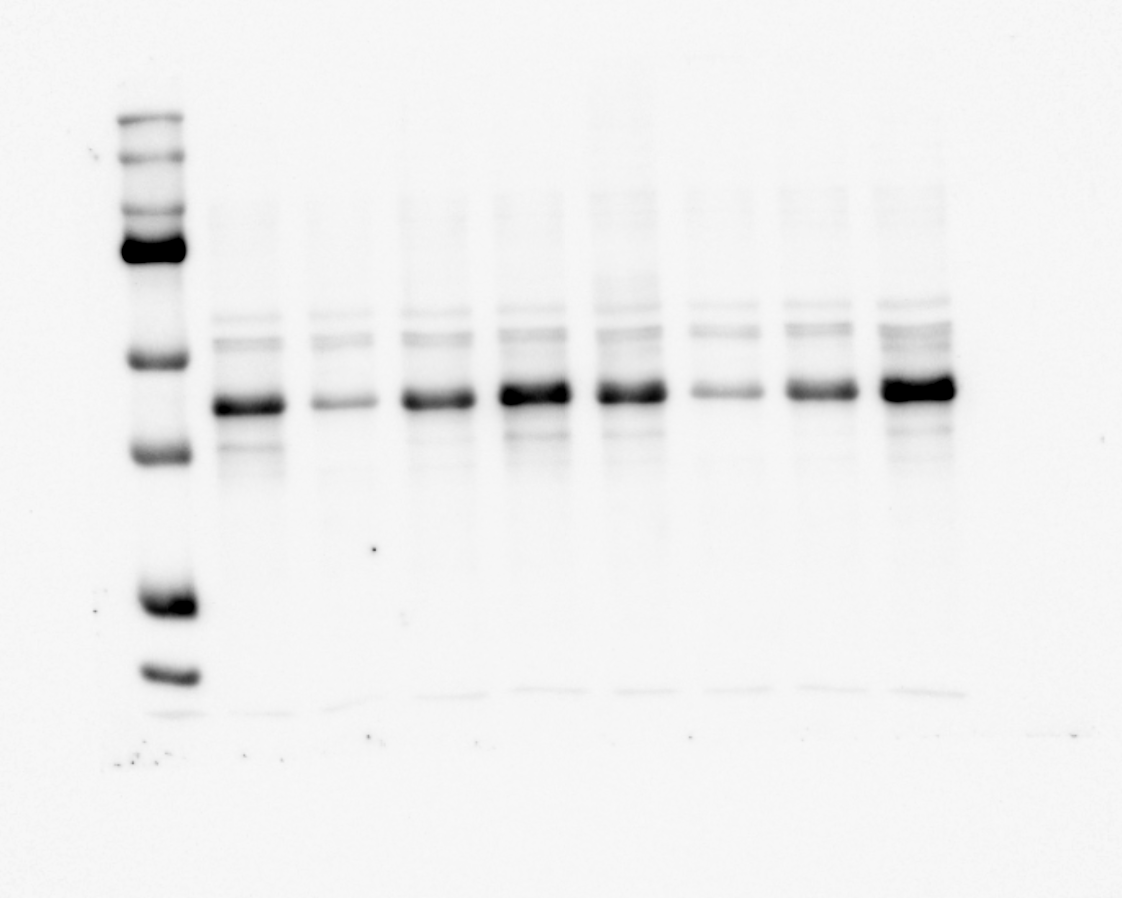

Supplement: Figure 1—source data 8. [file elife-102659-fig1-data8.zip › Figure 1 - Source data 8/Tyr279-phosphorylated GSK3a and Tyr216-phosphorylated GSK3b.tif]

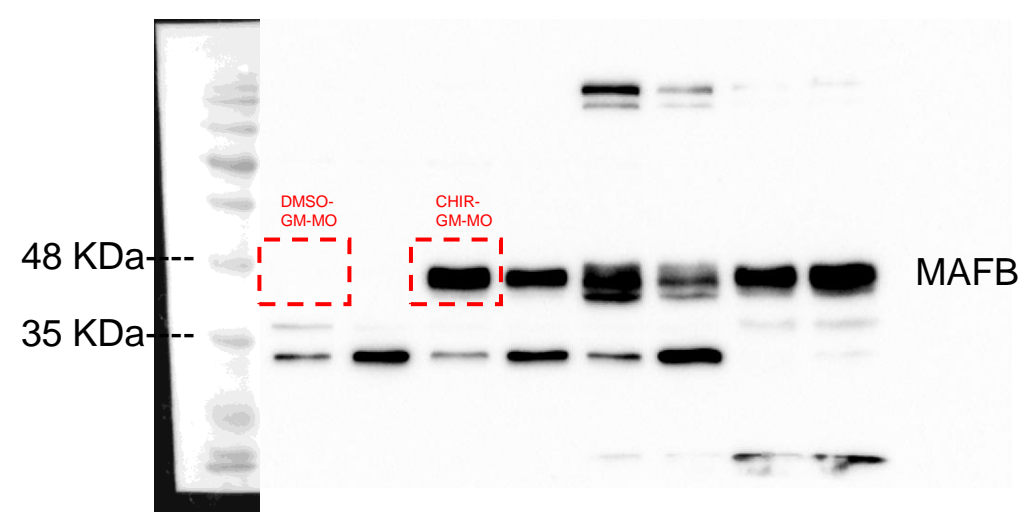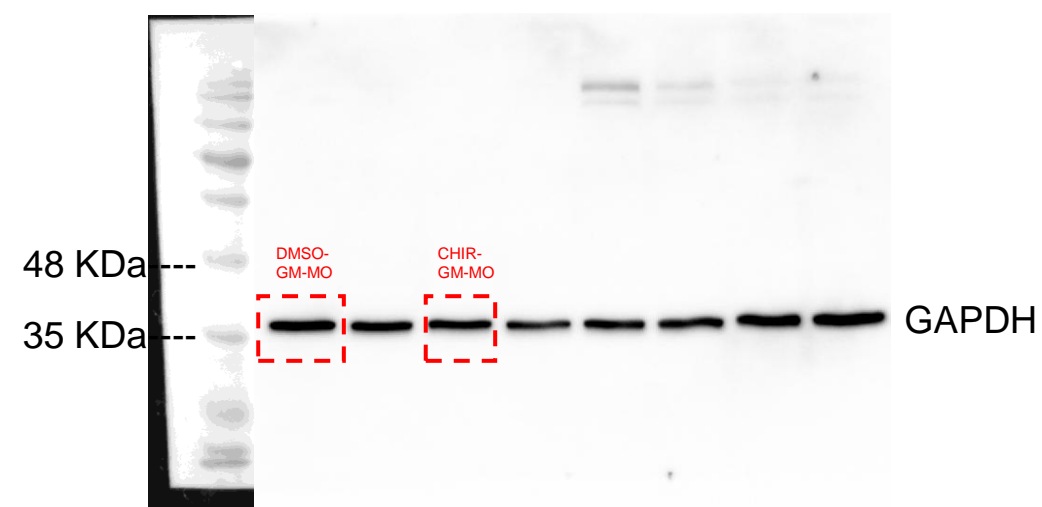

Figure 1E

Supplement: Figure 1—source data 10. [file elife-102659-fig1-data10.pdf]

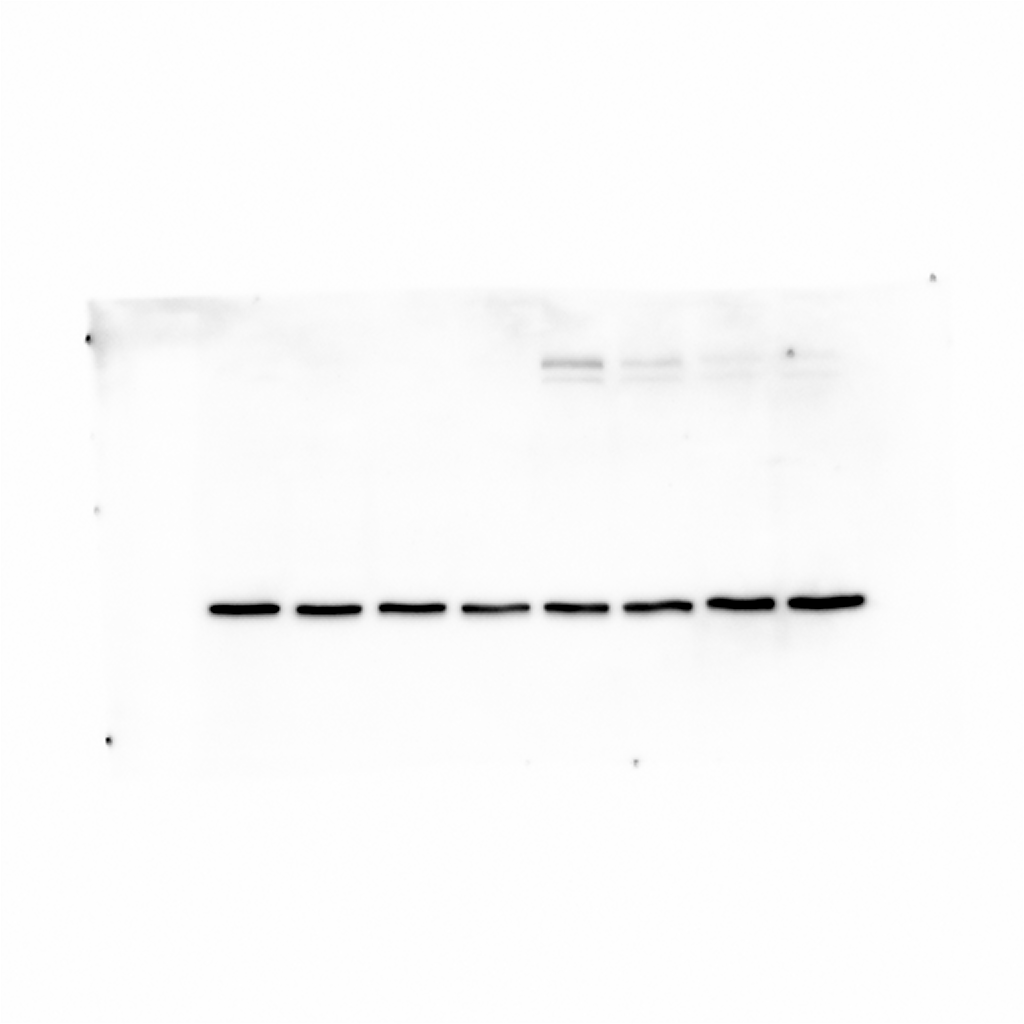

Supplement: Figure 1—source data 11. [file elife-102659-fig1-data11.zip › Figure 1 - Source data 11/GAPDH.Tif]

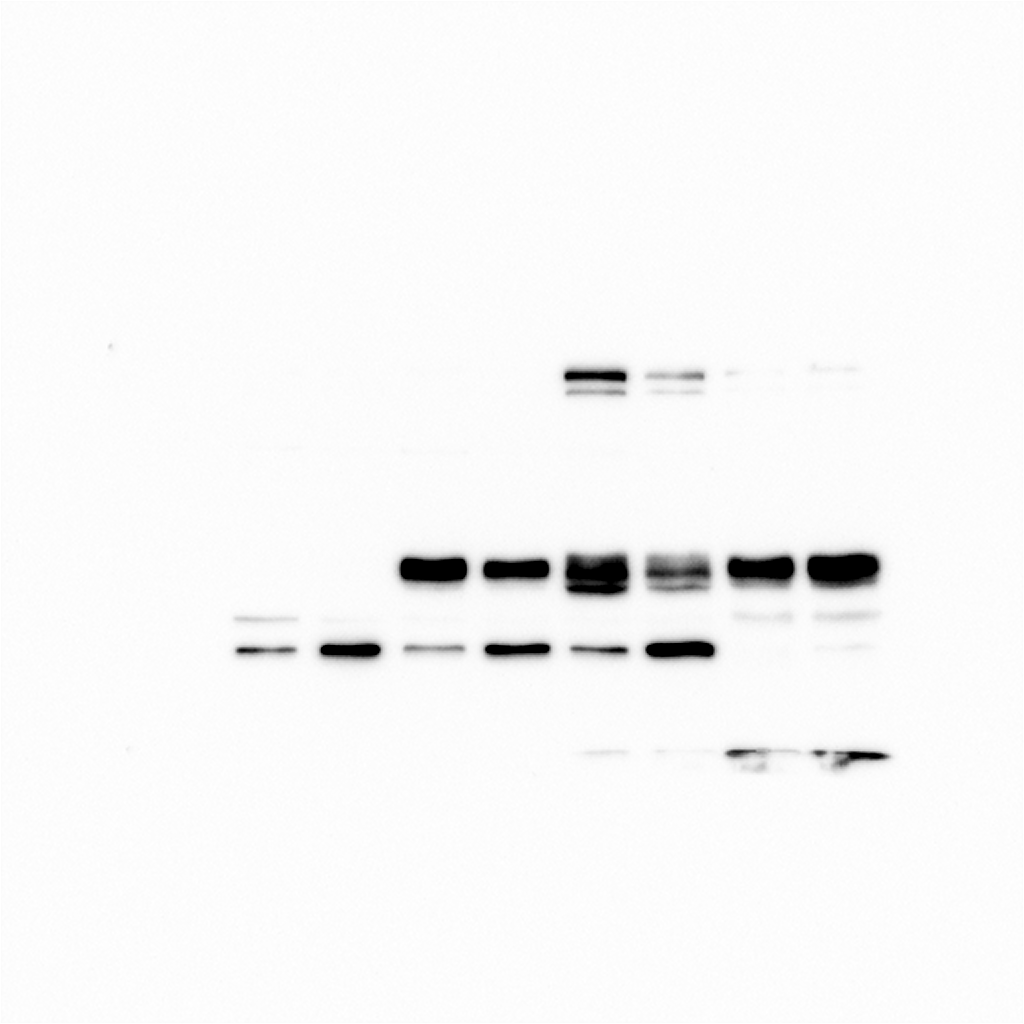

Supplement: Figure 1—source data 11. [file elife-102659-fig1-data11.zip › Figure 1 - Source data 11/MAFB.Tif]

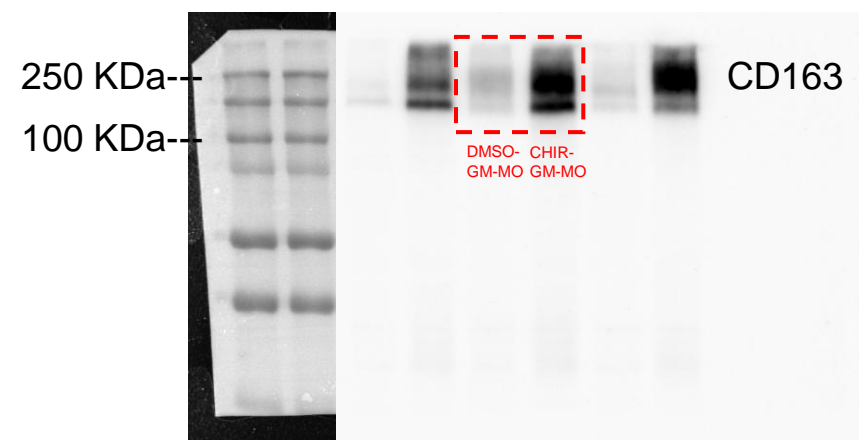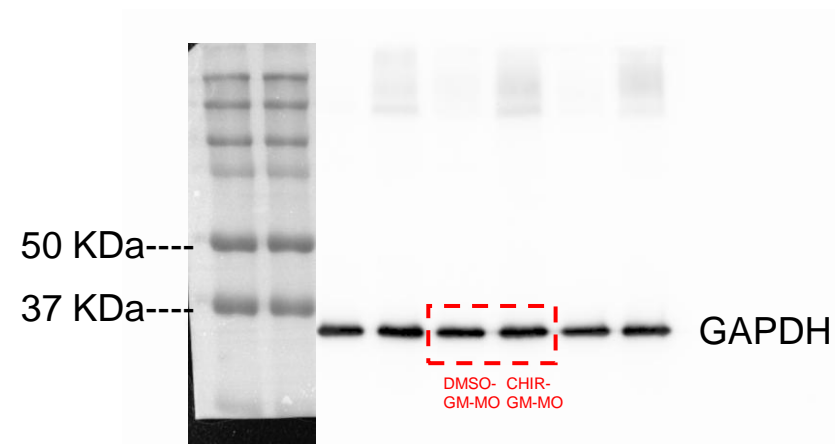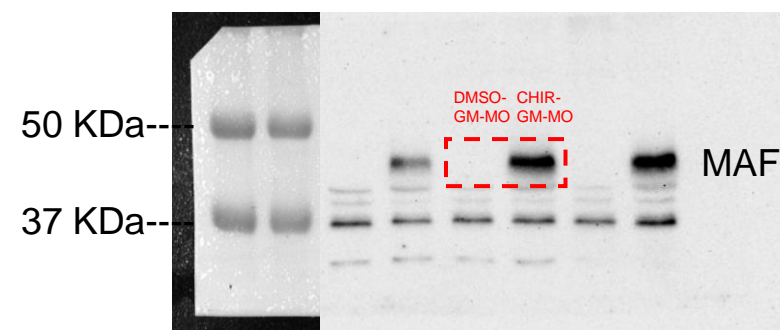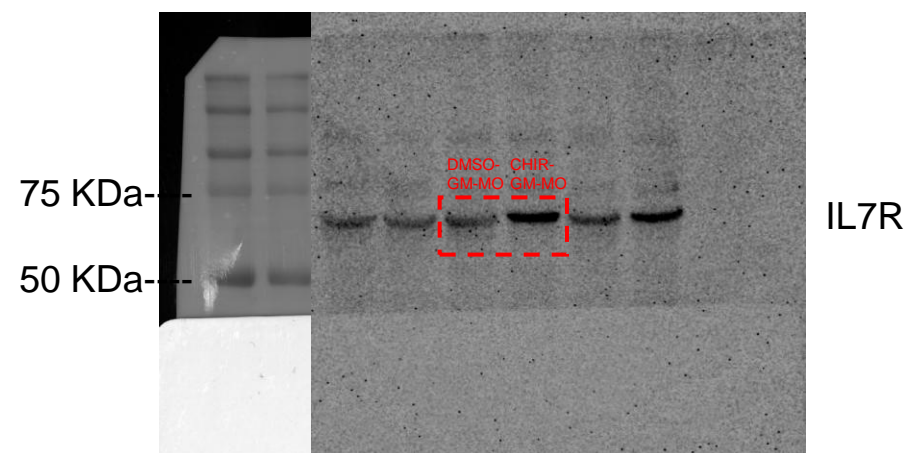

Figure 2E

Supplement: Figure 2—source data 3. [file elife-102659-fig2-data3.pdf]

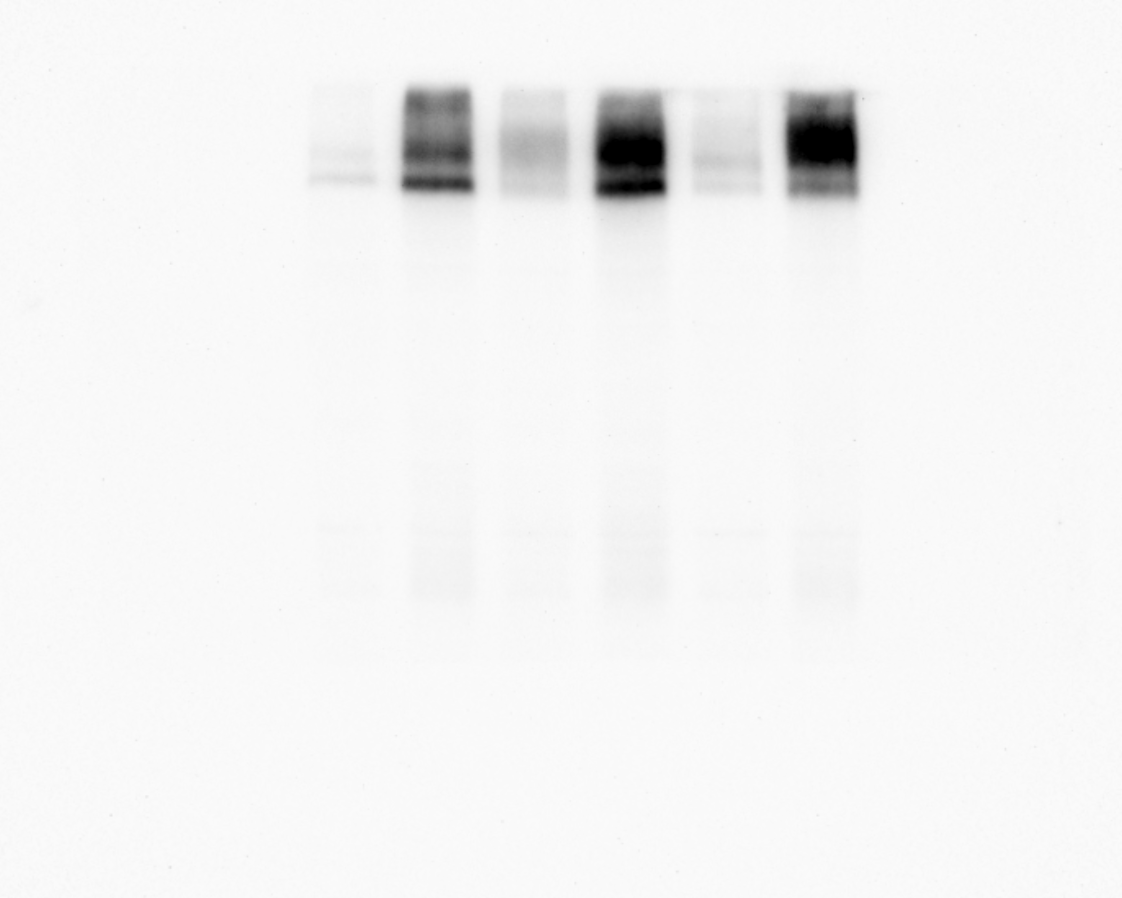

Supplement: Figure 2—source data 4. [file elife-102659-fig2-data4.zip › Figure 2 - Source data 4/CD163.tif]

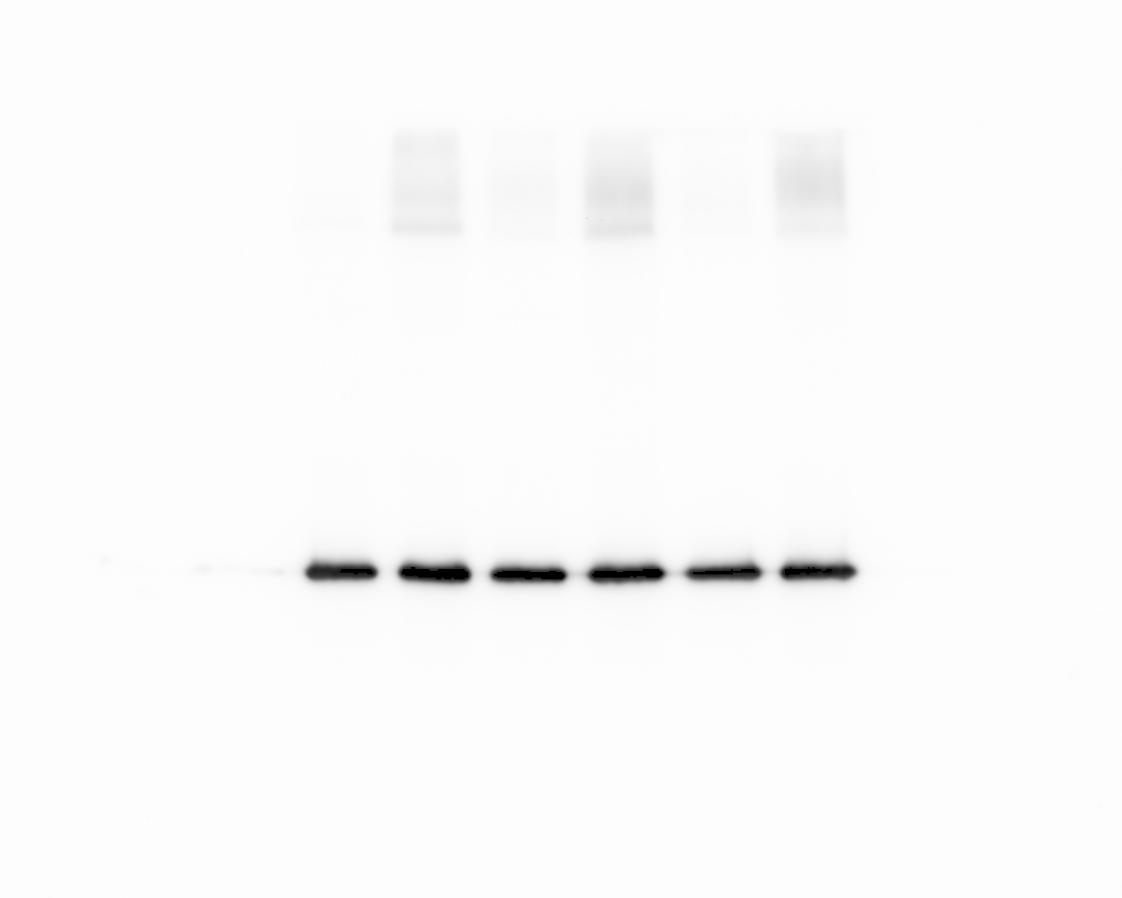

Supplement: Figure 2—source data 4. [file elife-102659-fig2-data4.zip › Figure 2 - Source data 4/GAPDH.tif]

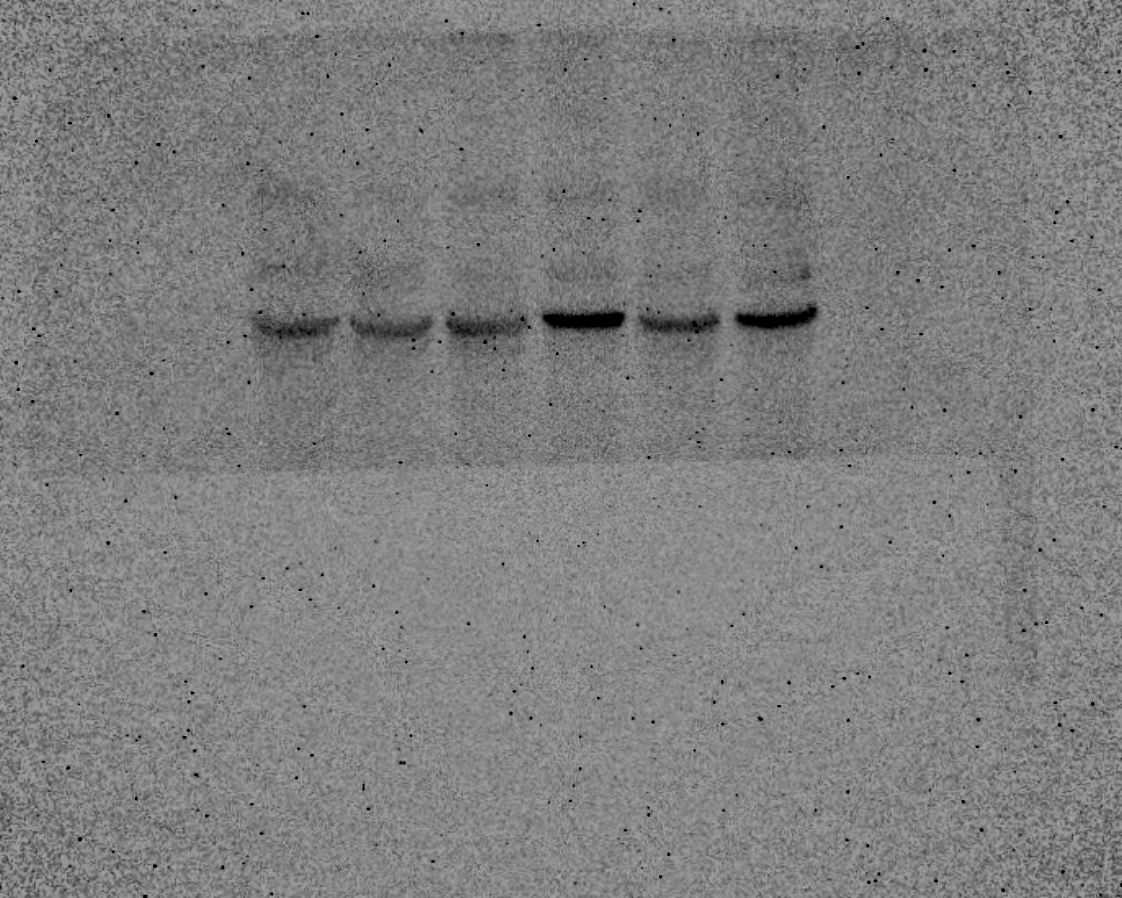

Supplement: Figure 2—source data 4. [file elife-102659-fig2-data4.zip › Figure 2 - Source data 4/IL7R.tif]

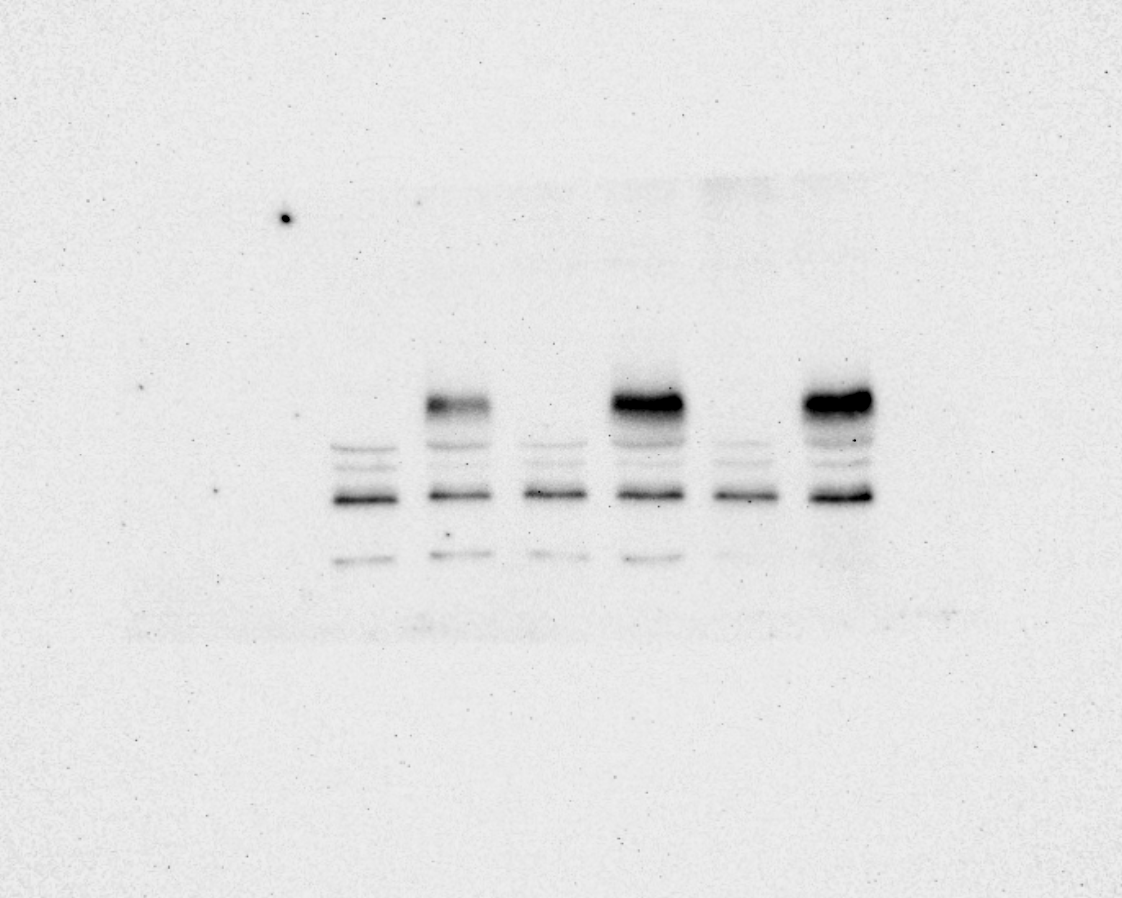

Supplement: Figure 2—source data 4. [file elife-102659-fig2-data4.zip › Figure 2 - Source data 4/MAF.tif]

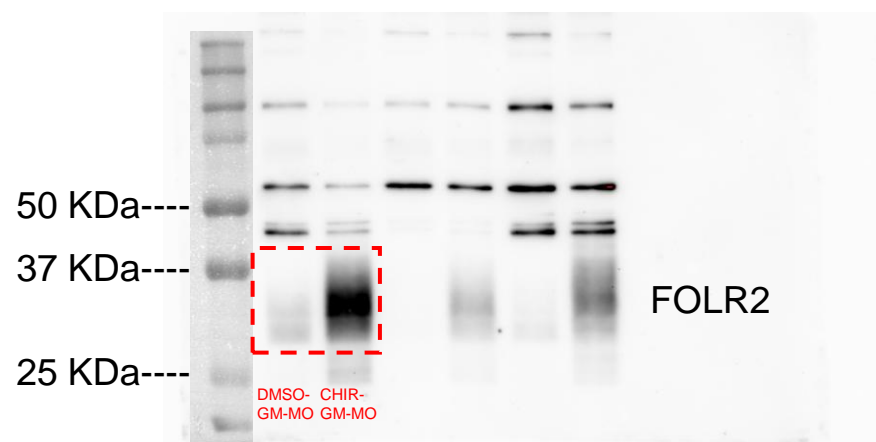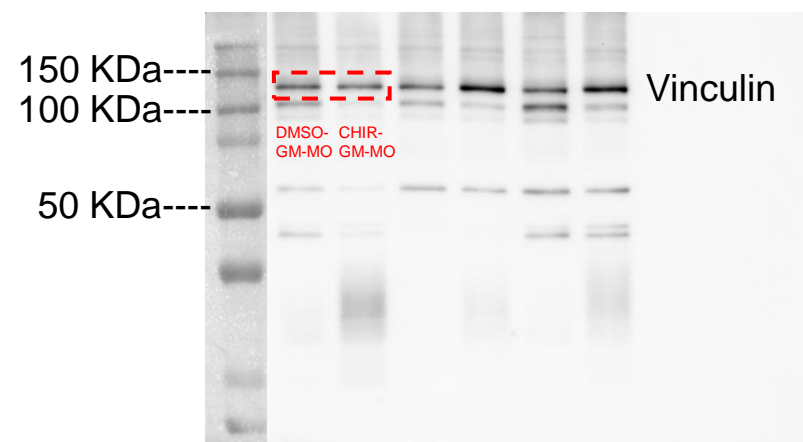

Figure 2F

Supplement: Figure 2—source data 6. [file elife-102659-fig2-data6.pdf]

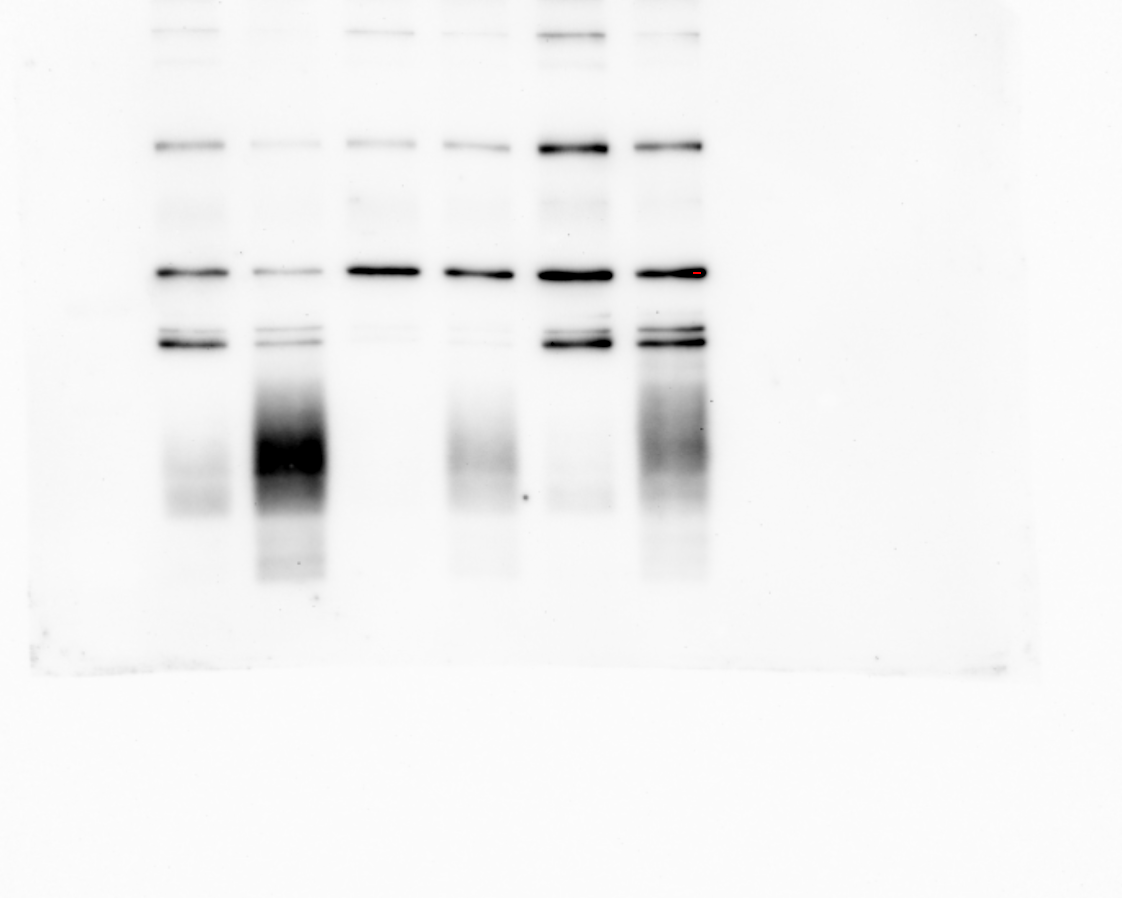

Supplement: Figure 2—source data 7. [file elife-102659-fig2-data7.zip › Figure 2 - Source data 7/FOLR2.tif]

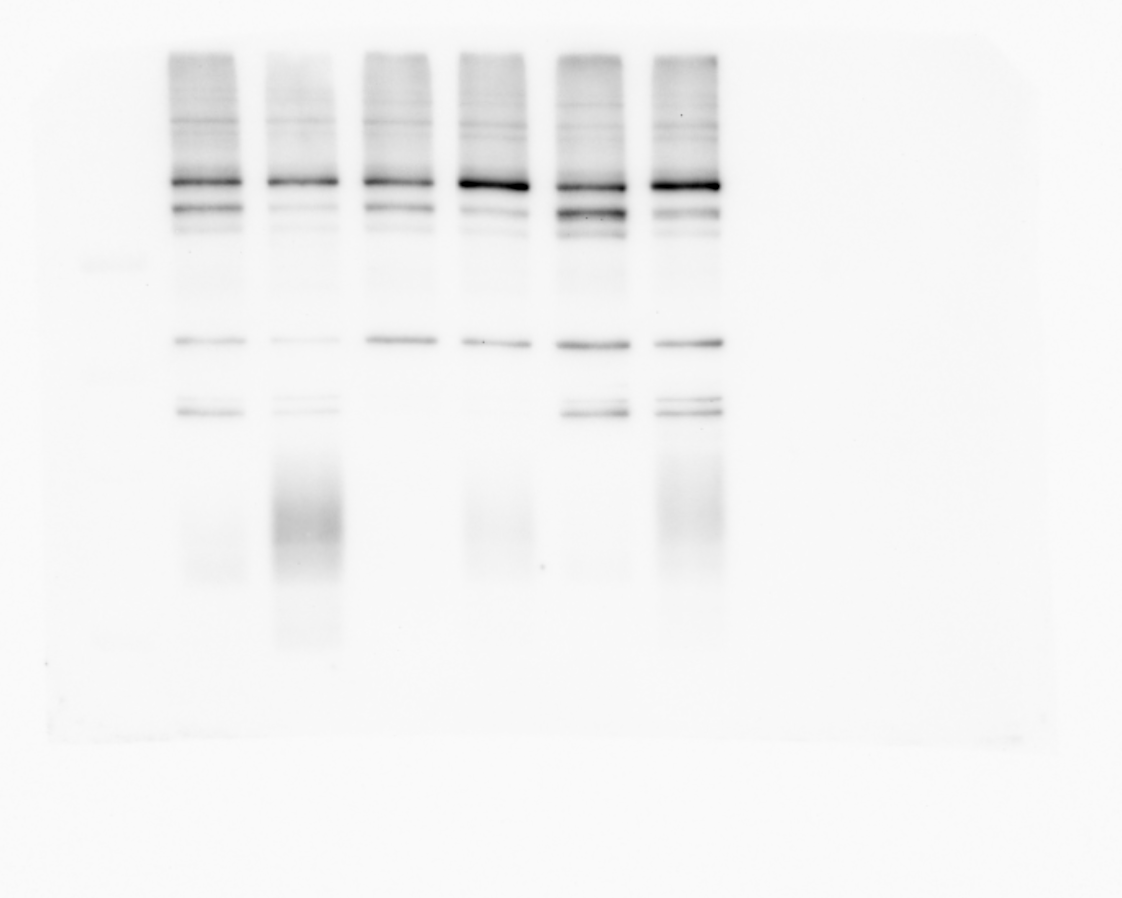

Supplement: Figure 2—source data 7. [file elife-102659-fig2-data7.zip › Figure 2 - Source data 7/Vinculin.tif]

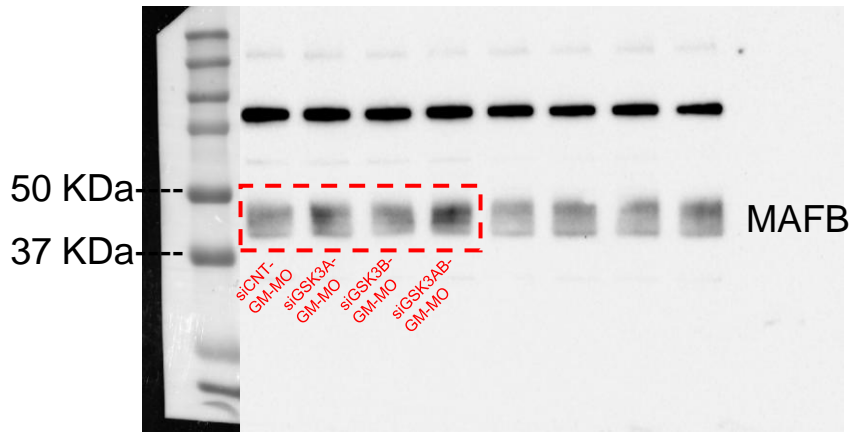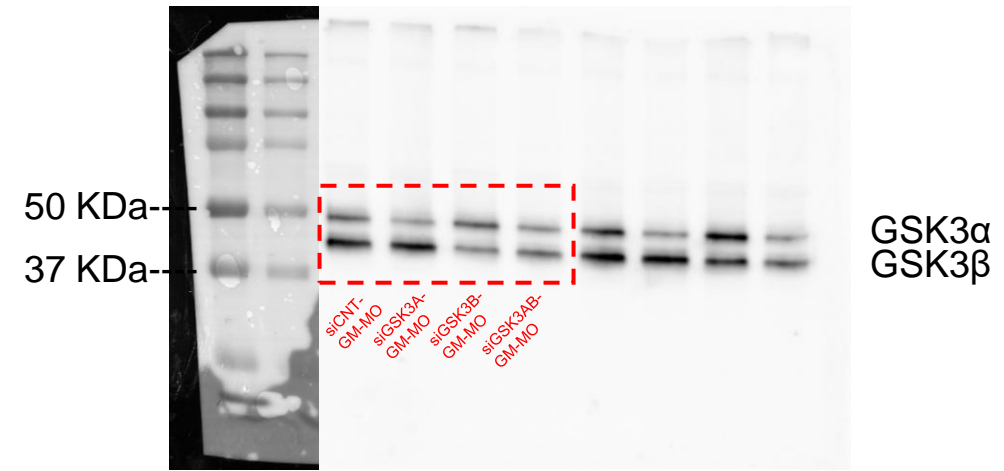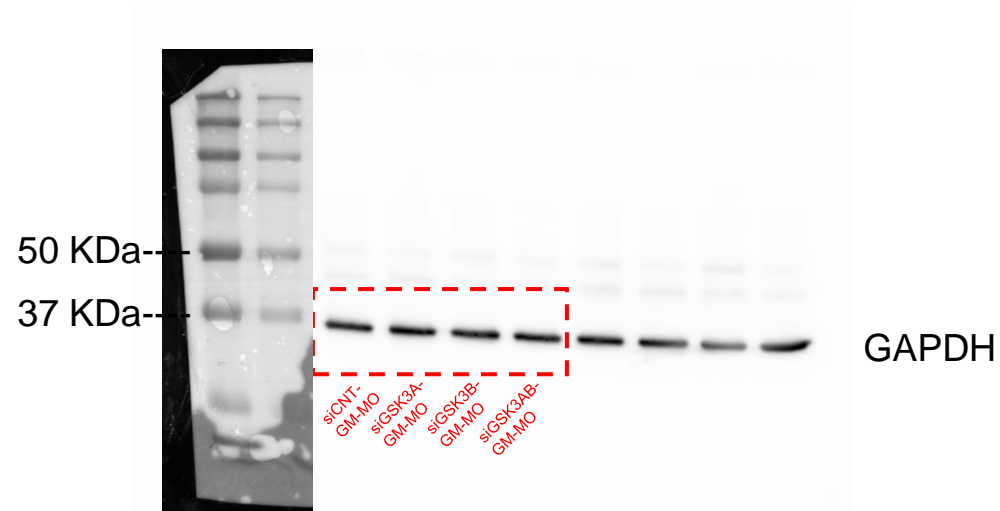

Figure 4B

Supplement: Figure 4—source data 1. [file elife-102659-fig4-data1.pdf]

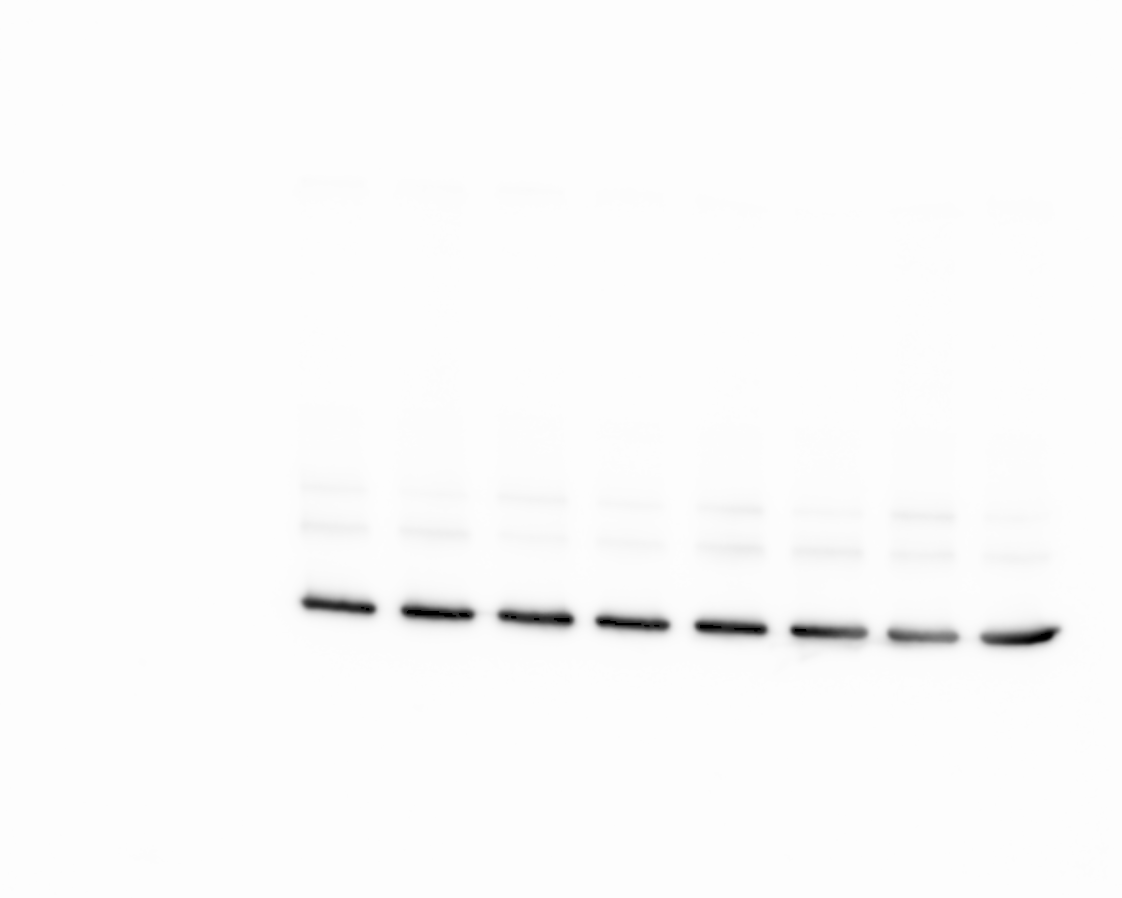

Supplement: Figure 4—source data 2. [file elife-102659-fig4-data2.zip › Figure 4 - Source data 2/GAPDH.tif]

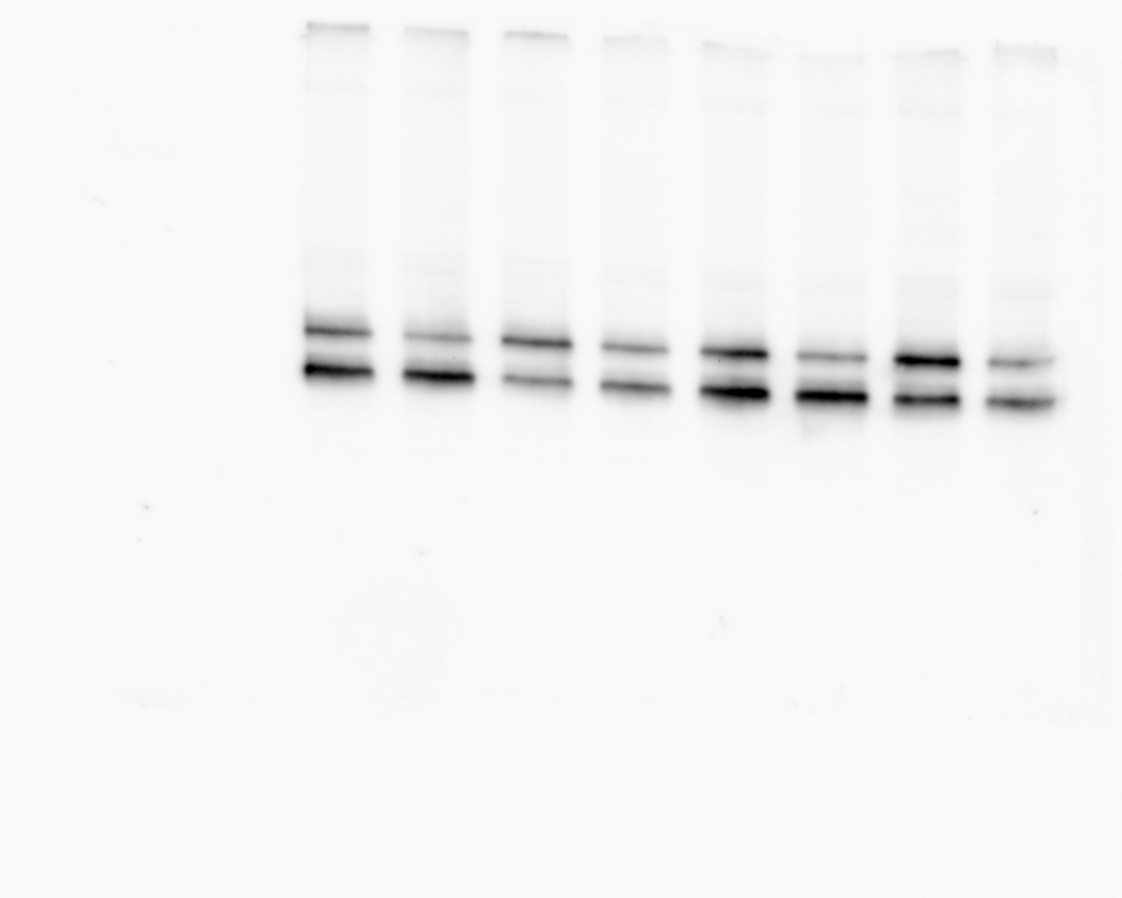

Supplement: Figure 4—source data 2. [file elife-102659-fig4-data2.zip › Figure 4 - Source data 2/GSK3a and GSK3b.tif]

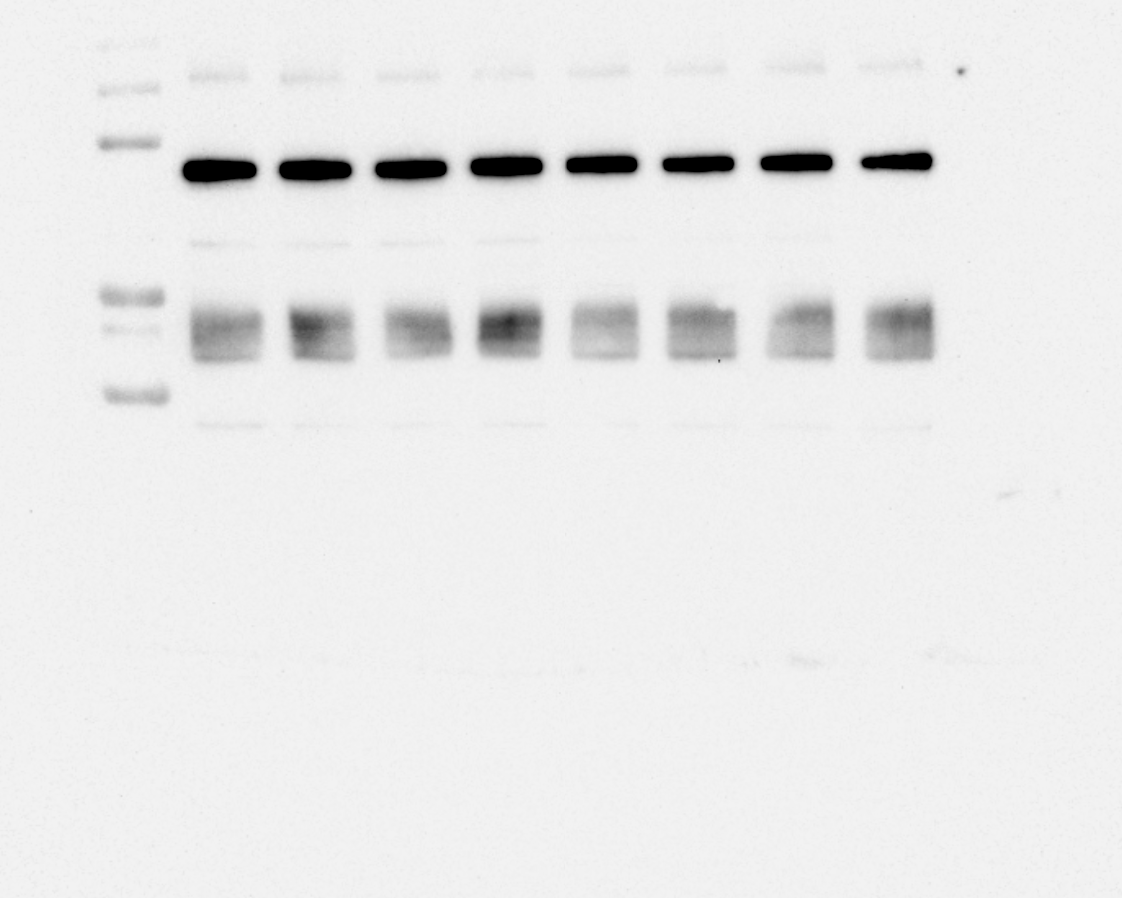

Supplement: Figure 4—source data 2. [file elife-102659-fig4-data2.zip › Figure 4 - Source data 2/MAFB.tif]

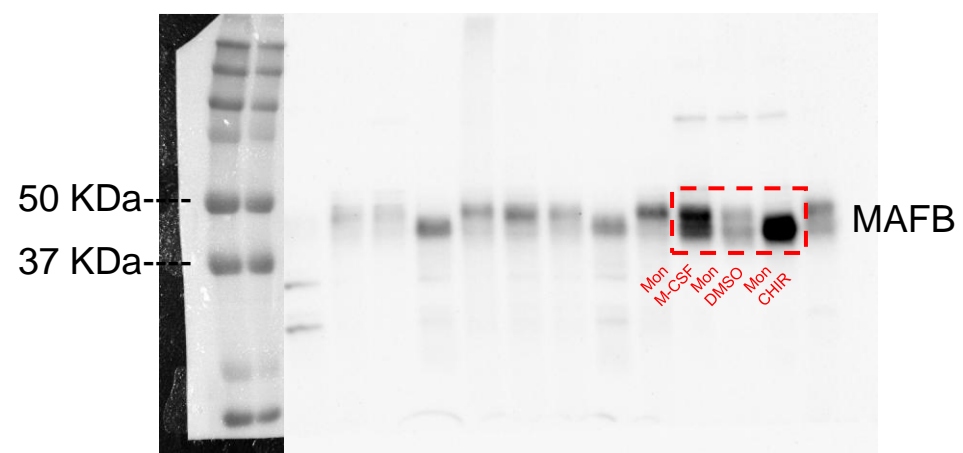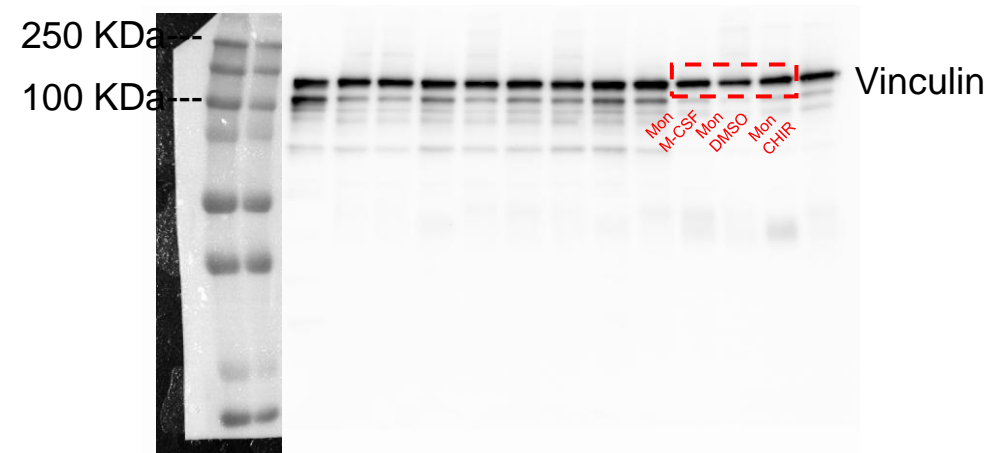

Figure 5B

Supplement: Figure 5—source data 1. [file elife-102659-fig5-data1.pdf]

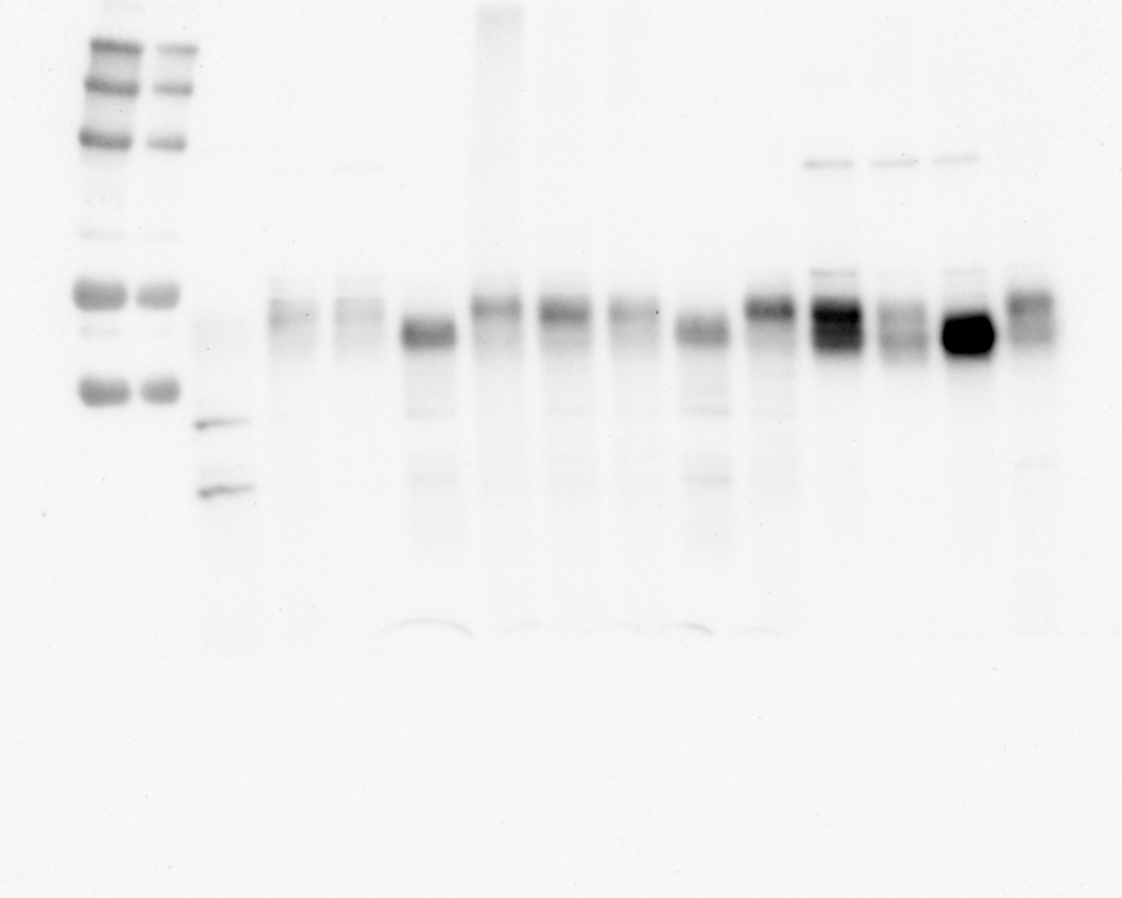

Supplement: Figure 5—source data 2. [file elife-102659-fig5-data2.zip › Figure 5 - Source data 2/MAFB.tif]

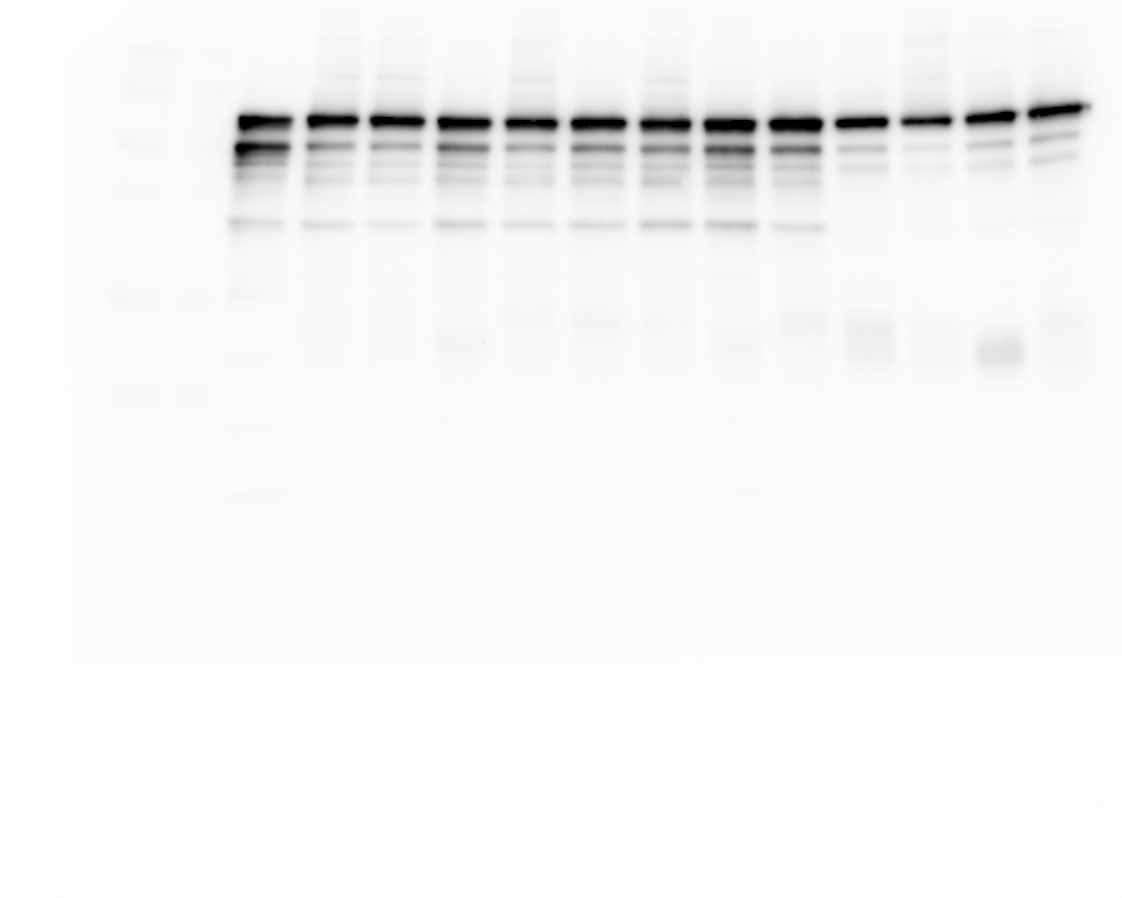

Supplement: Figure 5—source data 2. [file elife-102659-fig5-data2.zip › Figure 5 - Source data 2/vinculin.tif]

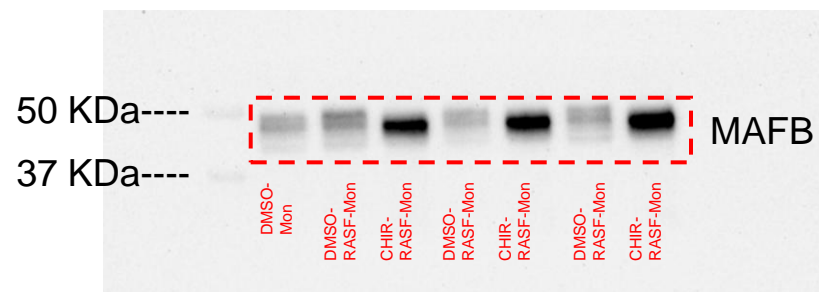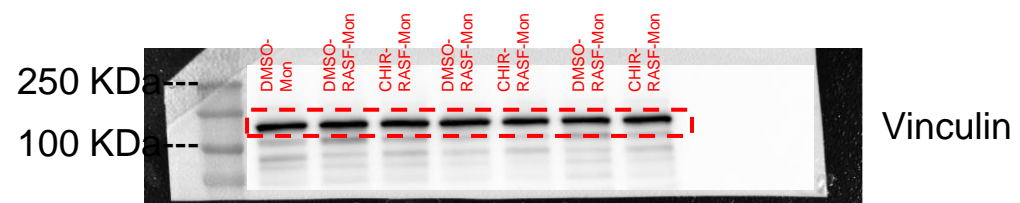

Figure 5I

Supplement: Figure 5—source data 5. [file elife-102659-fig5-data5.pdf]

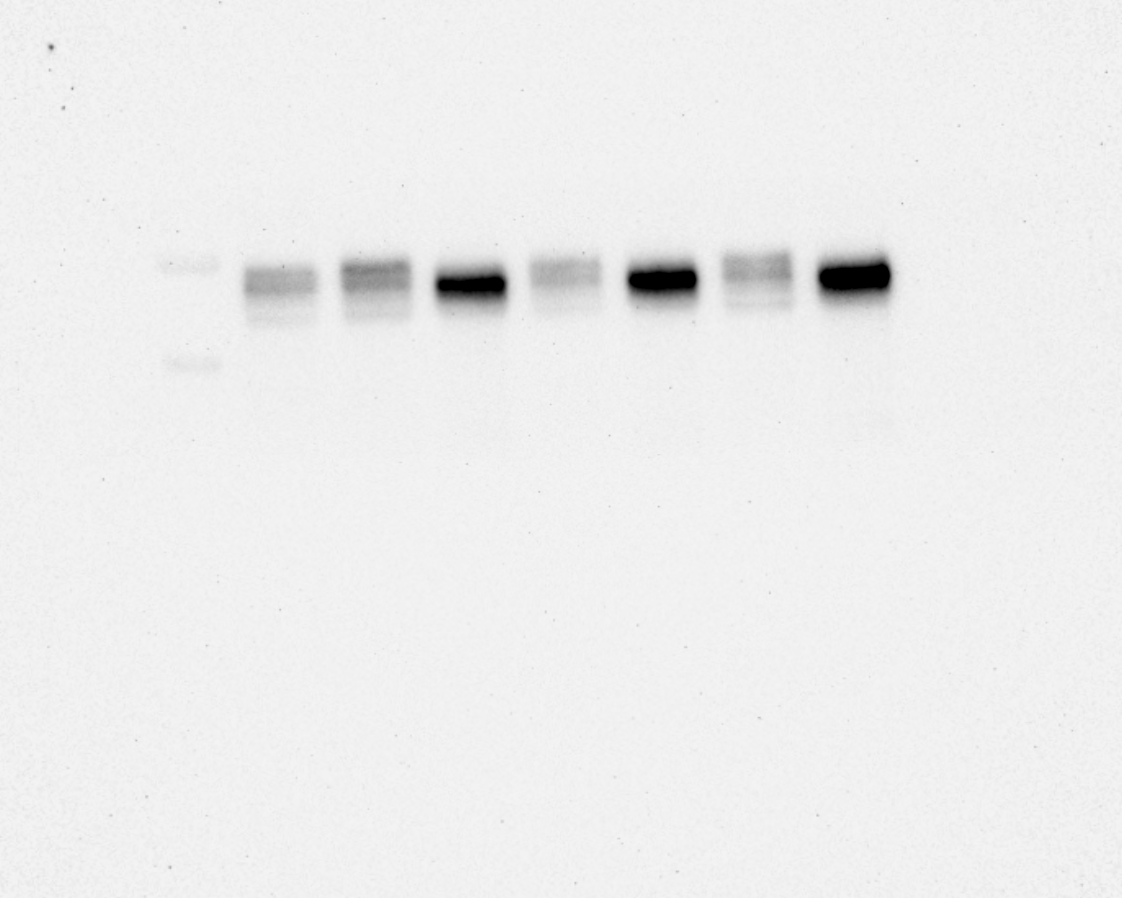

Supplement: Figure 5—source data 6. [file elife-102659-fig5-data6.zip › Figure 5 - Source data 6/MAFB.tif]

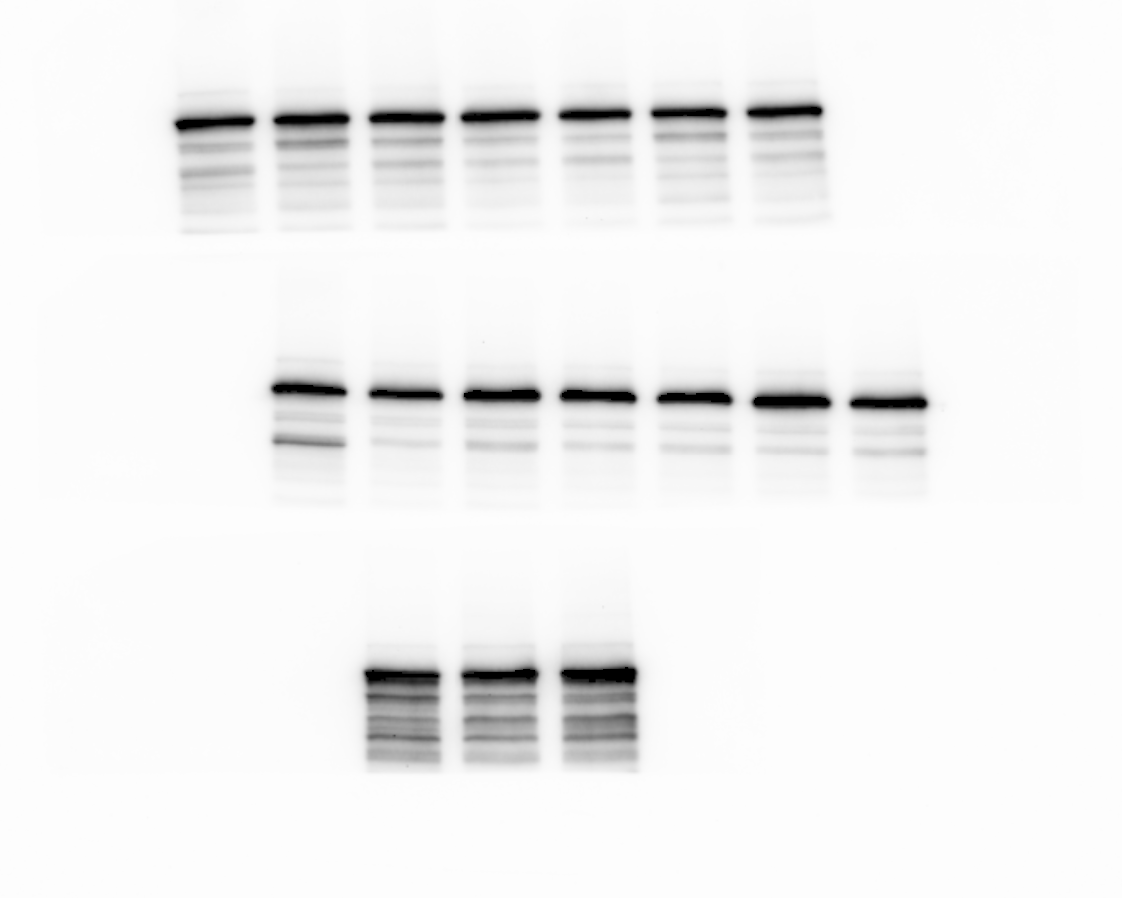

Supplement: Figure 5—source data 6. [file elife-102659-fig5-data6.zip › Figure 5 - Source data 6/Vinculin.tif]

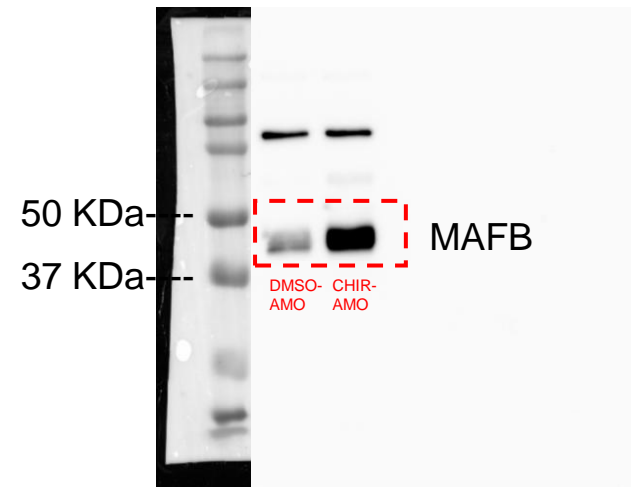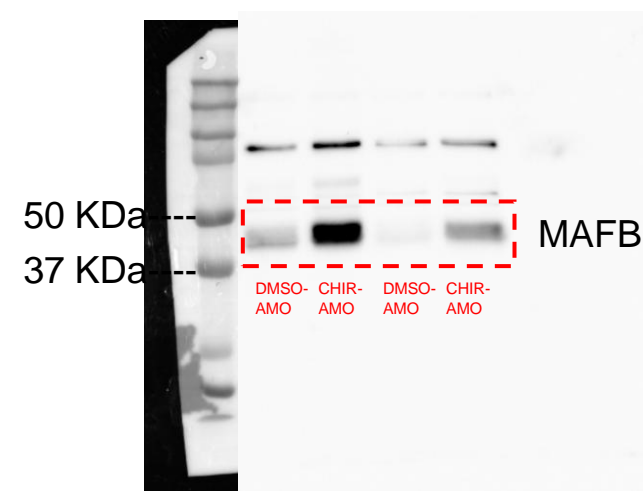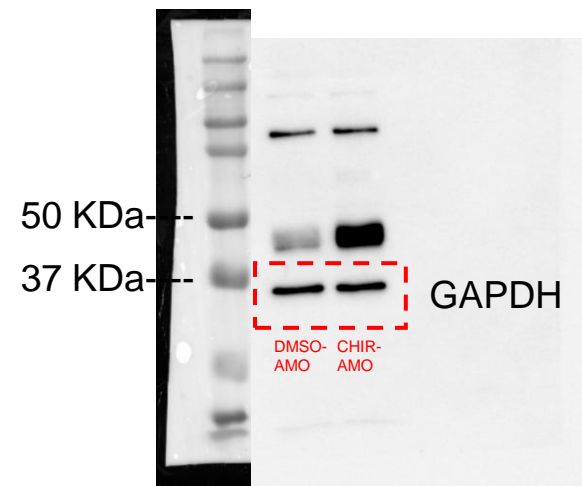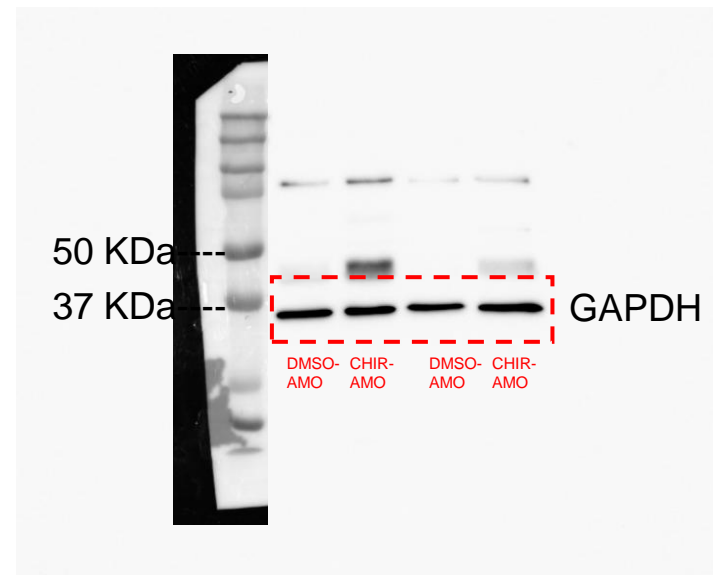

Figure 6F

Supplement: Figure 6—source data 1. [file elife-102659-fig6-data1.pdf]

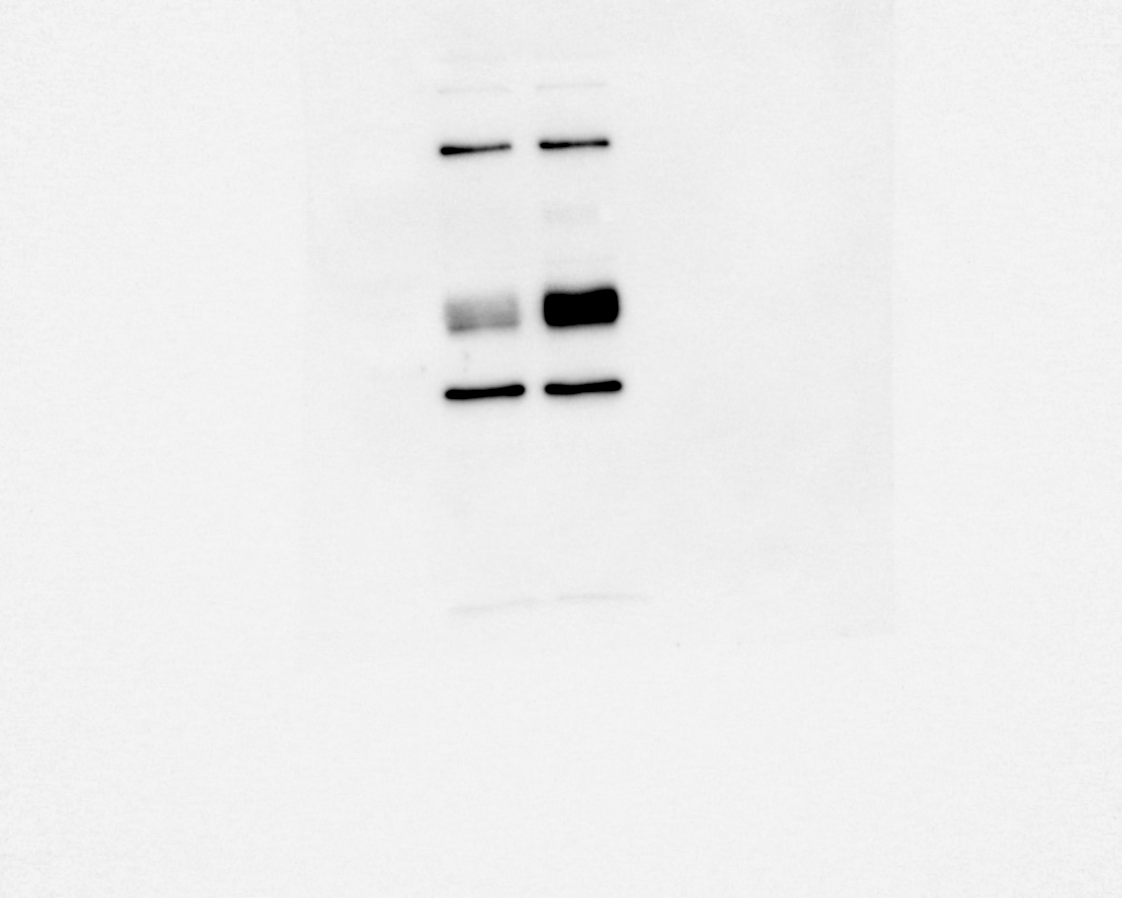

Supplement: Figure 6—source data 2. [file elife-102659-fig6-data2.zip › Figure 6 - Source data 2/GAPDH_AMO1.tif]

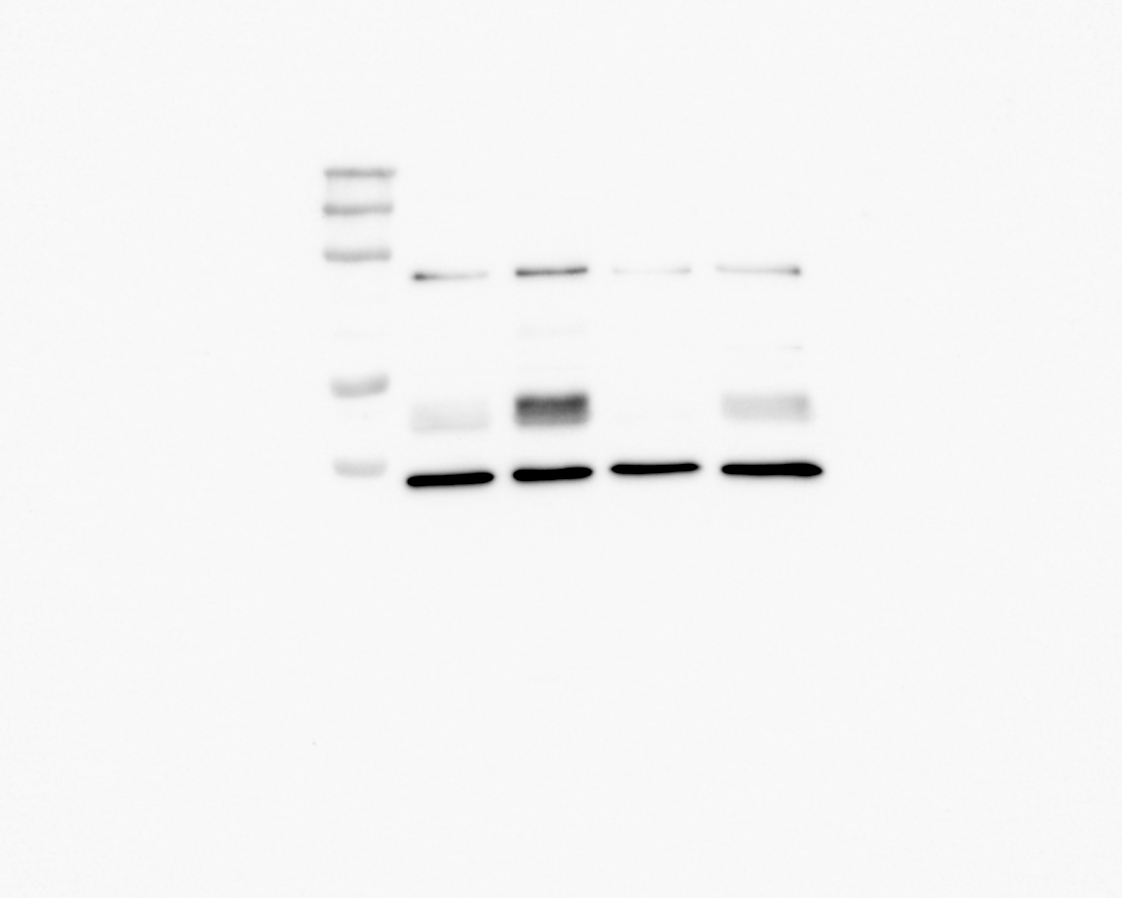

Supplement: Figure 6—source data 2. [file elife-102659-fig6-data2.zip › Figure 6 - Source data 2/GAPDH_AMO2 and AMO3.tif]

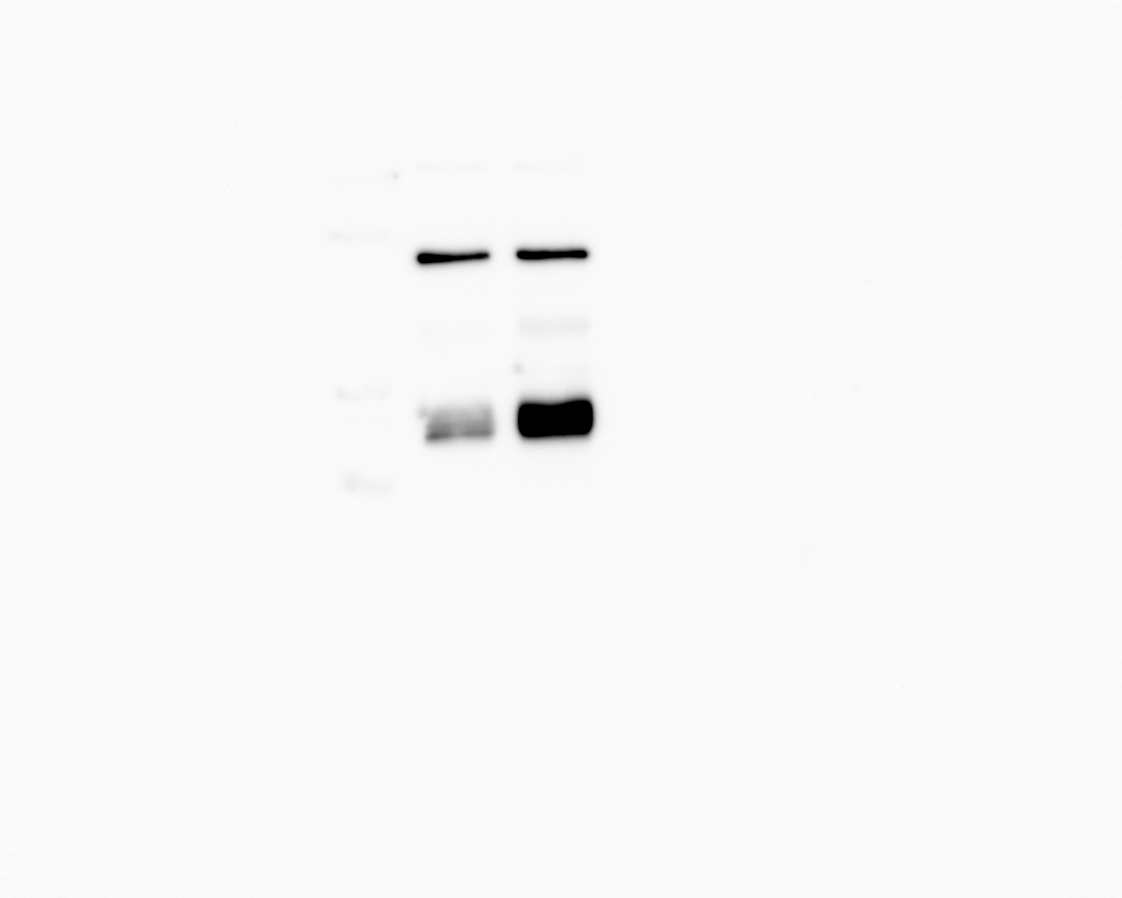

Supplement: Figure 6—source data 2. [file elife-102659-fig6-data2.zip › Figure 6 - Source data 2/MAFB_AMO1.tif]

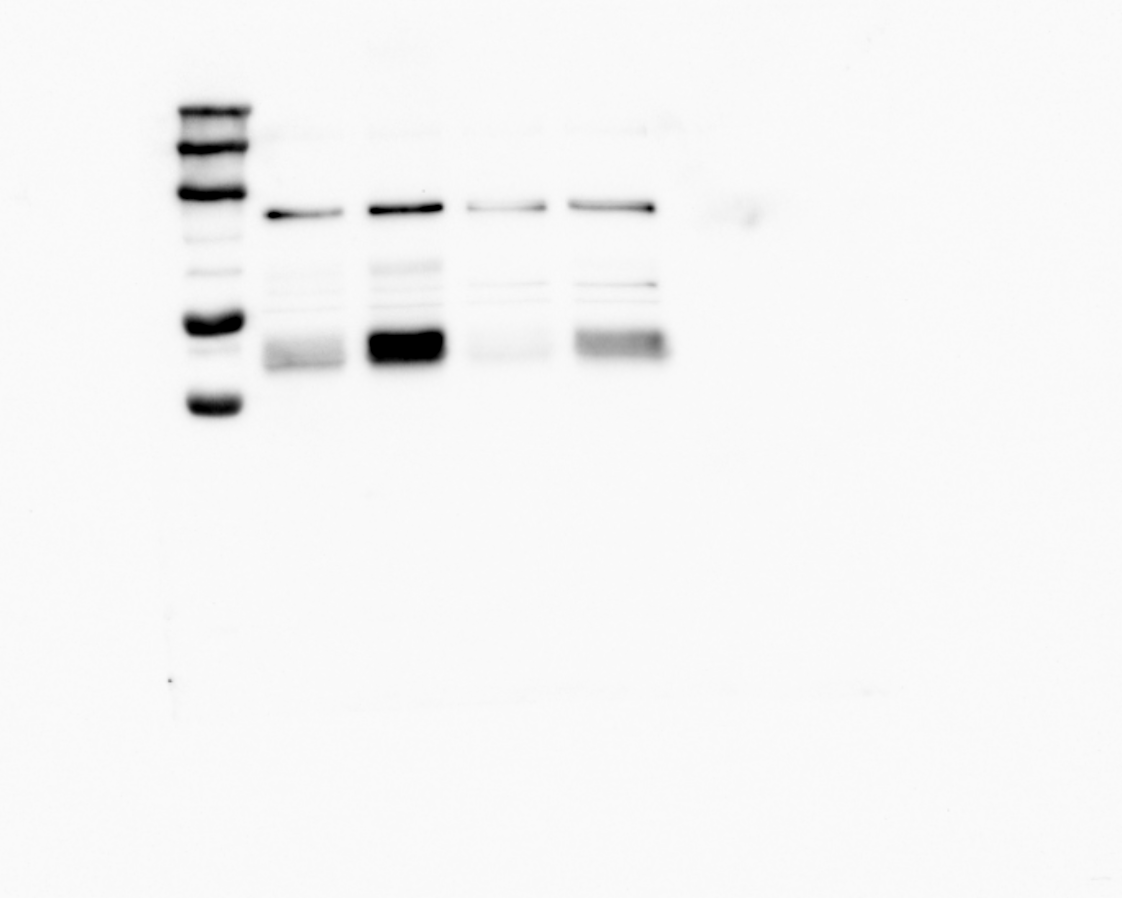

Supplement: Figure 6—source data 2. [file elife-102659-fig6-data2.zip › Figure 6 - Source data 2/MAFB_AMO2 and AMO3.tif]
