# Supplementary material for: Reprogramming of GM-CSF-dependent alveolar macrophages through GSK3 activity modulation
Source: eLife. 2025 May 14;14:RP102659. doi: 10.7554/eLife.102659 (PMC12077879; doi:10.7554/eLife.102659)
Supplement: Source code 1. [file elife-102659-code1.docx]

**Source code 1**

R code for single cell RNAseq analysis for Figure 7A-D, Sup Fig 3A-D, Sup Fig 4A-C

library(Seurat)

library(tidyverse)

library(ggplot2)

library(patchwork)

Healthy.1 <- ReadMtx(mtx = "GSM3660641_SC14NOR_matrix.mtx.gz",

features = "GSM3660641_SC14NOR_genes.tsv.gz",

cells = "GSM3660641_SC14NOR_barcodes.tsv.gz")

Healthy.2 <- ReadMtx(mtx = "GSM3660642_SC31NOR_matrix.mtx.gz",

features = "GSM3660642_SC31NOR_genes.tsv.gz",

cells = "GSM3660642_SC31NOR_barcodes.tsv.gz")

Healthy.3 <- ReadMtx(mtx = "GSM3660643_SC31DNOR_matrix.mtx.gz",

features = "GSM3660643_SC31DNOR_genes.tsv.gz",

cells = "GSM3660643_SC31DNOR_barcodes.tsv.gz")

Healthy.4 <- ReadMtx(mtx = "GSM3660644_SC45NOR_matrix.mtx.gz",

features = "GSM3660644_SC45NOR_genes.tsv.gz",

cells = "GSM3660644_SC45NOR_barcodes.tsv.gz")

Healthy.5 <- ReadMtx(mtx = "GSM3660645_SC56NOR_matrix.mtx.gz",

features = "GSM3660645_SC56NOR_genes.tsv.gz",

cells = "GSM3660645_SC56NOR_barcodes.tsv.gz")

Healthy.6 <- ReadMtx(mtx = "GSM3660646_SC59NOR_matrix.mtx.gz",

features = "GSM3660646_SC59NOR_genes.tsv.gz",

cells = "GSM3660646_SC59NOR_barcodes.tsv.gz")

Healthy.7 <- ReadMtx(mtx = "GSM3660647_SC155NORLOW_matrix.mtx.gz",

features = "GSM3660647_SC155NORLOW_genes.tsv.gz",

cells = "GSM3660647_SC155NORLOW_barcodes.tsv.gz")

Healthy.8 <- ReadMtx(mtx = "GSM3660648_SC156NORUP_matrix.mtx.gz",

features = "GSM3660648_SC156NORUP_genes.tsv.gz",

cells = "GSM3660648_SC156NORUP_barcodes.tsv.gz")

sdata.Healthy_1 <- CreateSeuratObject(Healthy.1, project = "Healthy_1")

sdata.Healthy_2 <- CreateSeuratObject(Healthy.2, project = "Healthy_2")

sdata.Healthy_3 <- CreateSeuratObject(Healthy.3, project = "Healthy_3")

sdata.Healthy_4 <- CreateSeuratObject(Healthy.4, project = "Healthy_4")

sdata.Healthy_5 <- CreateSeuratObject(Healthy.5, project = "Healthy_5")

sdata.Healthy_6 <- CreateSeuratObject(Healthy.6, project = "Healthy_6")

sdata.Healthy_7 <- CreateSeuratObject(Healthy.7, project = "Healthy_7")

sdata.Healthy_8 <- CreateSeuratObject(Healthy.8, project = "Healthy_8")

sdata.Healthy_1$type = "LUNG"

sdata.Healthy_2$type = "LUNG"

sdata.Healthy_3$type = "LUNG"

sdata.Healthy_4$type = "LUNG"

sdata.Healthy_5$type = "LUNG"

sdata.Healthy_6$type = "LUNG"

sdata.Healthy_7$type = "LUNG"

sdata.Healthy_8$type = "LUNG"

alldata <- merge(sdata.Healthy_1, c(sdata.Healthy_2, sdata.Healthy_3,sdata.Healthy_4, sdata.Healthy_5,sdata.Healthy_6,

sdata.Healthy_7,sdata.Healthy_8),

add.cell.ids = c("Healthy_1", "Healthy_2","Healthy_3","Healthy_4", "Healthy_5","Healthy_6",

"Healthy_7", "Healthy_8"))

rm(Healthy.1, Healthy.2, Healthy.3,Healthy.4, Healthy.5, Healthy.6,Healthy.7, Healthy.8,

sdata.Healthy_1, sdata.Healthy_2, sdata.Healthy_3,sdata.Healthy_4, sdata.Healthy_5,sdata.Healthy_6,

sdata.Healthy_7,sdata.Healthy_8)

gc()

modified_alldata <- alldata

modified_alldata <- PercentageFeatureSet(modified_alldata, "^MT-", col.name = "percent_mito")

VlnPlot(modified_alldata, features = c("nFeature_RNA", "nCount_RNA", "percent_mito"), ncol = 3)

modified_alldata <- subset(modified_alldata, subset = nFeature_RNA > 200 & nFeature_RNA < 6000

& nCount_RNA > 1000

& percent_mito < 15)

modified_alldata <- NormalizeData(modified_alldata)

modified_alldata <- FindVariableFeatures(modified_alldata, selection.method = "vst", nfeatures = 2000, verbose = FALSE)

top2000 <- head(VariableFeatures(modified_alldata), 2000)

modified_alldata <- modified_alldata[top2000]

modified_alldata <- JoinLayers(modified_alldata)

library(reticulate)

library(sceasy)

use_python("PATH/python.exe")

sc <- import("scanpy", convert = FALSE)

scvi <- import("scvi", convert = FALSE)

modified_alldata[["RNA"]] <- as(modified_alldata[["RNA"]], "Assay")

adata <- convertFormat(modified_alldata, from="seurat", to="anndata", main_layer="counts", drop_single_values=FALSE)

scvi$model$SCVI$setup_anndata(adata)

model = scvi$model$SCVI(adata, n_latent = 10L)

model$train()

latent = model$get_latent_representation()

latent <- as.matrix(latent)

rownames(latent) = colnames(modified_alldata)

modified_alldata[["scvi"]] <- CreateDimReducObject(embeddings = latent, key = "scvi_", assay = DefaultAssay(modified_alldata))

modified_alldata <- FindNeighbors(modified_alldata, dims = 1:10, reduction = "scvi")

modified_alldata <- FindClusters(modified_alldata, resolution = 1.2, reduction = "scvi")

modified_alldata <- RunUMAP(modified_alldata, dims = 1:10, reduction = "scvi", n.components = 2)

modified_alldata_markers <- FindAllMarkers(modified_alldata, only.pos = TRUE, min.pct = 0.25, logfc.threshold = 0.25)

write.csv(modified_alldata_markers, file = "Markers res1.2.csv")

p1 <- DimPlot(modified_alldata, group.by = "orig.ident", reduction = "scvi", label.size = 4, pt.size = 1.5)

p1

p2 <- DimPlot(modified_alldata, group.by = "seurat_clusters", reduction = "scvi", label.size = 4, pt.size = 1.5)

p2

p3 <- VlnPlot(modified_alldata, features = c("nFeature_RNA", "nCount_RNA", "percent_mito"),

group.by = "orig.ident", pt.size = 0)

p3

p4 <- FeaturePlot(modified_alldata, features = c("CD163","FABP4","LYVE1","FCN1","CD1C","VWF","CD3D","CD8A",

"IGHM","KLRD1","TPSAB1","SFTPC","AGER","FOXJ1","MUC5AC","COL1A1"), reduction = "scvi")

p4

clusters_to_merge <- c(0, 1, 2, 3, 4, 5, 6, 7, 8, 9, 10, 11, 12, 13, 14, 15, 16, 17, 18,

19, 20, 21, 22, 23, 24, 25,26, 27, 28, 29, 30, 31, 32, 33, 34, 35, 36)

modified_alldata$custom_cluster <- Idents(modified_alldata)

new_cluster_label_1 <- "Airway Goblet Cells"

new_cluster_label_2 <- "Alveolar Epithelial Type 1"

new_cluster_label_3 <- "Ciliated Cell Lung"

new_cluster_label_4 <- "DC"

new_cluster_label_5 <- "Endothelial Cell Lung"

new_cluster_label_6 <- "Lymphocyte"

new_cluster_label_7 <- "Macrophage"

new_cluster_label_8 <- "Mast Cell"

new_cluster_label_9 <- "Monocyte"

new_cluster_label_10 <- "NK"

new_cluster_label_11 <- "Fibroblast"

new_cluster_label_12 <- "Pneumocyte Lung Type II"

modified_alldata$custom_cluster <- factor(

modified_alldata$custom_cluster,

levels = c(levels(modified_alldata$custom_cluster), new_cluster_label_1, new_cluster_label_2,

new_cluster_label_3, new_cluster_label_4,new_cluster_label_5, new_cluster_label_6,

new_cluster_label_7, new_cluster_label_8,new_cluster_label_9, new_cluster_label_10,

new_cluster_label_11, new_cluster_label_12))

modified_alldata$custom_cluster[modified_alldata$custom_cluster %in% c(26)] <- new_cluster_label_1

modified_alldata$custom_cluster[modified_alldata$custom_cluster %in% c(31)] <- new_cluster_label_2

modified_alldata$custom_cluster[modified_alldata$custom_cluster %in% c(17)] <- new_cluster_label_3

modified_alldata$custom_cluster[modified_alldata$custom_cluster %in% c(25)] <- new_cluster_label_4

modified_alldata$custom_cluster[modified_alldata$custom_cluster %in% c(15, 20, 21, 33)] <- new_cluster_label_5

modified_alldata$custom_cluster[modified_alldata$custom_cluster %in% c(2, 10, 12, 30, 36)] <- new_cluster_label_6

modified_alldata$custom_cluster[modified_alldata$custom_cluster %in% c(0, 1, 3, 5, 7, 8, 11, 16, 19, 23, 27, 28, 32, 35)] <- new_cluster_label_7

modified_alldata$custom_cluster[modified_alldata$custom_cluster %in% c(24)] <- new_cluster_label_8

modified_alldata$custom_cluster[modified_alldata$custom_cluster %in% c(4, 6, 29)] <- new_cluster_label_9

modified_alldata$custom_cluster[modified_alldata$custom_cluster %in% c(9, 22)] <- new_cluster_label_10

modified_alldata$custom_cluster[modified_alldata$custom_cluster %in% c(14, 34)] <- new_cluster_label_11

modified_alldata$custom_cluster[modified_alldata$custom_cluster %in% c(13, 18)] <- new_cluster_label_12

DimPlot(modified_alldata, group.by = "custom_cluster", reduction = "scvi", label = T,

label.size = 2.5, repel = T, pt.size = 1.5)

p5 <- DimPlot(modified_alldata, group.by = "seurat_clusters", reduction = "scvi", label.size = 4, pt.size = 1.5)

p5

### Macrophage reintegration

Macrophage = subset(modified_alldata,idents = c('0','1','3','4','5','6','7','8','11','16','19','23','27','28','29',

'32','35'))

DefaultAssay(Macrophage) <- "RNA"

Macrophage <- NormalizeData(Macrophage)

Macrophage <- FindVariableFeatures(Macrophage, selection.method = "vst",nfeatures = 2000, verbose = FALSE)

top2000 <- head(VariableFeatures(Macrophage), 2000)

Macrophage <- Macrophage[top2000]

sc <- import("scanpy", convert = FALSE)

scvi <- import("scvi", convert = FALSE)

Macrophage[["RNA"]] <- as(Macrophage[["RNA"]], "Assay")

adata_macrophage <- convertFormat(Macrophage, from = "seurat", to = "anndata", main_layer = "counts", drop_single_values = FALSE)

scvi$model$SCVI$setup_anndata(adata_macrophage)

model_macrophage <- scvi$model$SCVI(adata_macrophage, n_latent = 10L)

model_macrophage$train()

latent_macrophage <- model_macrophage$get_latent_representation()

latent_macrophage <- as.matrix(latent_macrophage)

rownames(latent_macrophage) <- colnames(Macrophage)

Macrophage[["scvi"]] <- CreateDimReducObject(embeddings = latent_macrophage, key = "scvi_", assay = DefaultAssay(Macrophage))

Macrophage <- FindNeighbors(Macrophage, dims = 1:10, reduction = "scvi")

Macrophage <- FindClusters(Macrophage, resolution = 0.6)

Macrophage <- RunUMAP(Macrophage, dims = 1:10, reduction = "scvi", n.components = 2)

pm1 <- DimPlot(Macrophage, reduction = "scvi", label = T)

pm1

pm2 <- DimPlot(Macrophage, reduction = "scvi", group.by = "orig.ident", label = F, pt.size = 1.5)

pm2

pm3 <- DimPlot(Macrophage, reduction = "scvi", group.by = "seurat_clusters", label = F, pt.size = 1.5)

pm3

pm4 <- FeaturePlot(Macrophage, features = c("FCN1","FABP4","MARCO", "INHBA","FOLR2","LYVE1","LGMN","CD163",

"TYMS","MKI67", "TOP2A", "NUSAP1"),

min.cutoff = "q10", reduction = "scvi")

pm4

pm5 <- VlnPlot(Macrophage, features = c("FCN1","FABP4","MARCO", "INHBA","FOLR2","LYVE1","LGMN","CD163",

"TYMS","MKI67", "TOP2A", "NUSAP1"),

group.by = "RNA_snn_res.0.6",

stack = TRUE, flip = TRUE)

pm5

Macrophage_markers <- FindAllMarkers(Macrophage, only.pos = TRUE, min.pct = 0.25, logfc.threshold = 0.25, reduction = "scvi")

write.csv(Macrophage_markers, file = "Macrophage Markers res0.6.csv")

clusters_to_merge <- c(0, 1, 2, 3, 4, 5, 6, 7, 8, 9, 10, 11, 12, 13, 14, 15)

Macrophage$custom_cluster <- Idents(Macrophage)

new_cluster_label_1 <- "Monocyte"

new_cluster_label_2 <- "AM"

new_cluster_label_3 <- "IM"

new_cluster_label_4 <- "Prolif.M"

Macrophage$custom_cluster <- factor(

Macrophage$custom_cluster,

levels = c(levels(Macrophage$custom_cluster), new_cluster_label_1, new_cluster_label_2,

new_cluster_label_3, new_cluster_label_4))

Macrophage$custom_cluster[Macrophage$custom_cluster %in% c(4, 7, 12)] <- new_cluster_label_1

Macrophage$custom_cluster[Macrophage$custom_cluster %in% c(0, 2, 5, 9, 11, 13)] <- new_cluster_label_2

Macrophage$custom_cluster[Macrophage$custom_cluster %in% c(1, 3, 6, 8, 10, 14)] <- new_cluster_label_3

Macrophage$custom_cluster[Macrophage$custom_cluster %in% c(15)] <- new_cluster_label_4

pm6 <-DimPlot(Macrophage, group.by = "custom_cluster", reduction = "scvi")

pm6

pm7 <-DotPlot(Macrophage, features = c("MAFB","IL10", "LGMN","CD163","FOLR2","LYVE1","SPP1","CCL18","CCL2",

"MS4A6A","CXCL8","CYP1B1","IL4I1","LDLR","CD300E","BATF"),

group.by = "custom_cluster", dot.min = 0, dot.scale = 25,cols = c("blue","red")) + RotatedAxis()

pm7

pm8 <-DotPlot(Macrophage, features = c("FABP4","INHBA","GSN","FBP1",

"PPARG","CXCL5","AXL","AQP3","CLEC4E",

"ABCG1","CITED2"),

group.by = "custom_cluster", dot.min = 0, dot.scale = 25, cols = c("blue","red")) + RotatedAxis()

pm8

bulk <- AggregateExpression(Macrophage, group.by = c("custom_cluster","orig.ident"),return.seurat = F)

bulk <- bulk$RNA

bulk1 <- as.data.frame(bulk)

write.csv(bulk1, file = "pseudoRNAseq Macrophages.csv")
